# Enhancing stroke rehabilitation with whole-hand haptic rendering: development and clinical usability evaluation of a novel upper-limb rehabilitation device

**DOI:** 10.1186/s12984-024-01439-1

**Published:** 2024-09-27

**Authors:** Raphael Rätz, François Conti, Irène Thaler, René M. Müri, Laura Marchal-Crespo

**Affiliations:** 1 Motor Learning and Neurorehabilitation Laboratory, ARTORG Center for Biomedical Engineering Research, University of Bern, Bern, Switzerland; 2 Force Dimension, Nyon, Switzerland; 3 Department of Neurology, University Neurorehabilitation, University Hospital Bern (Inselspital), University of Bern, Bern, Switzerland; 4 Department of Cognitive Robotics, Delft University of Technology, Delft, The Netherlands; 5 Department of Rehabilitation Medicine, University Medical Center Rotterdam, Rotterdam, The Netherlands

**Keywords:** Neurorehabilitation, Stroke, Sensorimotor, Robotic, Haptic rendering, Clinical-driven, Usability, Upper-limb, Grasping, Serious game

## Abstract

**Introduction:**

There is currently a lack of easy-to-use and effective robotic devices for upper-limb rehabilitation after stroke. Importantly, most current systems lack the provision of somatosensory information that is congruent with the virtual training task. This paper introduces a novel haptic robotic system designed for upper-limb rehabilitation, focusing on enhancing sensorimotor rehabilitation through comprehensive haptic rendering.

**Methods:**

We developed a novel haptic rehabilitation device with a unique combination of degrees of freedom that allows the virtual training of functional reach and grasp tasks, where we use a physics engine-based haptic rendering method to render whole-hand interactions between the patients’ hands and virtual tangible objects. To evaluate the feasibility of our system, we performed a clinical mixed-method usability study with seven patients and seven therapists working in neurorehabilitation. We employed standardized questionnaires to gather quantitative data and performed semi-structured interviews with all participants to gain qualitative insights into the perceived usability and usefulness of our technological solution.

**Results:**

The device demonstrated ease of use and adaptability to various hand sizes without extensive setup. Therapists and patients reported high satisfaction levels, with the system facilitating engaging and meaningful rehabilitation exercises. Participants provided notably positive feedback, particularly emphasizing the system’s available degrees of freedom and its haptic rendering capabilities. Therapists expressed confidence in the transferability of sensorimotor skills learned with our system to activities of daily living, although further investigation is needed to confirm this.

**Conclusion:**

The novel haptic robotic system effectively supports upper-limb rehabilitation post-stroke, offering high-fidelity haptic feedback and engaging training tasks. Its clinical usability, combined with positive feedback from both therapists and patients, underscores its potential to enhance robotic neurorehabilitation.

**Supplementary Information:**

The online version contains supplementary material available at 10.1186/s12984-024-01439-1 .

## Introduction

Stroke is a major contributor to long-term disability and mortality worldwide, with over twelve million incidents annually [1]. Amongst the consequences of surviving a stroke, the loss of upper-limb functions such as reaching, grasping, and fine object manipulation—critical to carrying out activities of daily living (ADL)—are particularly prevalent, affecting around 55% - 85% of stroke survivors [2–7]. To promote recovery, patients should actively engage [8] in highly intense [8–11] task-specific exercises [10], which require one-to-one constant involvement of clinical personnel. This leads to a heavy burden on society and healthcare institutions. The expected increase in stroke incidents due to an aging society [12, 13] and the foreseen global health staff shortage [14] calls for a profound transformation in current clinical practice towards more sustainable, accessible, efficient, and effective stroke rehabilitation.

Robotic devices have become increasingly popular in neurorehabilitation research as they hold the potential to support therapists in providing highly intense, effective, and motivating rehabilitation training with minimal physical effort from the therapists [15–17]. To enable the use of robotic devices to their full potential, they are often combined with gamified virtual training tasks [18], which have been reported to enhance patient enjoyment [19]. Moreover, the provision of real-time performance feedback allows therapists to adapt the training to the patient’s individual needs [20]. When compared to dose-matched conventional therapy, some studies point to improved outcomes with robotic interventions [17, 21, 22], while a large body of research indicates that robotic therapy is currently non-inferior to conventional interventions [15, 23–26].

Vast efforts are currently put into further enhancing the benefits of rehabilitation robotics. An emerging line of research is to not only focus on motor functions but also provide meaningful somatosensory information during robot-assisted training [16, 27–30]. This information, acquired through our skin and muscle mechanoreceptors during physical interactions with tangible (real-life or virtual) objects, is crucial for successful movement execution, as highlighted by a myriad of studies. For example, Pettypiece et al. reported that even in a predominantly visual task, somatosensory information greatly influences motor performance [31], and Scott et al. found that the sensorimotor system can be described as an optimal feedback controller where the state estimation depends on the availability of sensory information [32]. The importance of somatosensory information is also exemplified by the syndromes of clinical conditions such as tactile apraxia [33] or sensory ataxia [34]. Although some stroke survivors with somatosensory impairments (observed in more than half of stroke survivors [35]) succeed in relearning fine object manipulation to a certain degree—e.g., by compensating with vision [36]—somatosensory impairments such as limited tactile sensibility are still a considerable cause of inconvenience in daily living for affected patients [37–39]. Indeed, it has been suggested that the recovery of somatosensory impairments might even be imperative for the full recovery of a paretic upper limb [40].

Therefore, it is recommended that meaningful sensory information should be incorporated in robotic training, for example, by physically representing the interaction forces with tangible objects from virtual environments—a process known as haptic rendering [16, 28, 41, 42]. A few robotic devices have been developed for haptic rendering in upper-limb neurorehabilitation. They are engineered to accurately generate forces from virtual training tasks and provide low resistance to self-initiated movements in the absence of such forces. This can be achieved through mechanical characteristics such as low inertia, high backdrivability, low backlash, and/or appropriate control methods. Their mechanical structure can vary, influencing their application in haptic rendering and clinical practicability [16].

Within grounded arm exoskeletons, i.e., those solutions where the robot joints are coincident with the anatomical arm and shoulder joints, we can find solutions that promote haptic rendering during the haptic manipulation of virtual objects, e.g., ALEx-RS [43], ARMin [44], and ANYexo [45, 46]. However, while grounded exoskeletons generally provide high levels of support and can control individual joints of the patients, they come at the cost of high complexity and rather complicated setup due to the necessary joint alignments. In contrast, grounded end-effector devices only interact at an end-point of the patient, e.g., hand or wrist. They tend to offer more flexibility and easier setup together with inherently low mechanical inertia as they usually incorporate the actuators in the base. Popular designs include planar five-bar manipulanda with parallel kinematics—e.g., MIT-Manus [47], InMotion^®^ ARM/HAND [48], WristBot [49] or the device of Qian et al. [50]—, devices with a combination of two linear axes to achieve planar movements—e.g., H-Man [51] and ArmMotus M2 Pro [52]—, and serial kinematic designs that offer arm movements in three-dimensional space—e.g., Burt^®^ [53] and ArmMotus^™^ EMU [54].

Such devices that predominantly target the proximal joints often either use a simple cylindrical handle or lack hand-related interfaces to interact with virtual objects. Yet, when grasping and manipulating objects, the distal body parts, such as the wrist and hand, also play a crucial role in gathering somatosensory information. Thus efforts have been made in developing devices that provide haptic rendering at more distal joints [55], such as grounded devices—e.g., HEXORR-II [56], ReHapticKnob [57], HandyBot [58], FINGER [59], the portable hand trainer from Van Damme et al. [60, 61], or the OpenWrist [62]—and more wearable devices like gloves or hand and wrist exoskeletons—e.g., [63–65]. The latter usually only generate forces within the (distal) attachment points. Thus, their application for haptic rendering can be limited due to the lack of force generation on proximal joints.

When reviewing the literature, we found a relatively limited number of robotic solutions that provide haptic rendering in both arm and hand. This is a limitation since functional reach and grasp movements are typically composed of coordinated movements of proximal and distal joints [66–68]. We only found a few examples in literature that allow virtual training of reaching and grasping while also providing haptic rendering that targets hand functions. For example, Buongionrno et al. combined the ALEx arm exoskeleton [69] with wrist and hand exoskeletons, resulting in a 12 DoF device, and presented haptic rendering of a virtual stick in a box, although reaching out and grasping the stick was not reported [70]. A reach and grasp task with haptic cues was presented by Loureiro et al. with the nine DoF Gentle/G system [71]. In the reachMAN2, three DoF were combined to train simple reach and grasp movements [72]. Finally, the CyberTeam^®^ system consists of a five DoF glove and a six DoF robotic base [73]. However, these devices still tend to be highly complex and/or bulky, hampering their potential clinical applicability. To our best knowledge, only the Gentle/G has been tested with patients [74].

Moreover, the vast majority of haptic devices in neurorehabilitation follow classical haptic rendering approaches, where either virtual walls [75] or one or multiple predetermined interaction points in the form of spherical colliders (i.e., virtual representation to compute collisions) are used to compute interaction forces [43, 46]. These methods can only simulate interactions at predetermined locations on objects and thus fail to represent arbitrary hand-object interactions. This might result in visuo-haptic incongruencies during virtual haptic reach and grasp exercises, which might lead to hindered motor performance [76], and increased cognitive load [77, 78]. Importantly, more realistic whole-hand interactions, where the haptic rendering reflects the entire visual hand representation, may lead to more natural interactions with virtual objects [79], enhancing the ecological validity of the training and potentially facilitating the transfer of the acquired skills to ADL [80].

Hence, we have identified a clear need for a device for the training of coordinated proximal and distal movements alongside high-fidelity whole-hand haptic rendering that provides meaningful haptic information during reaching and grasping. Importantly, the device should be simple to use yet sophisticated enough to accomplish the aforementioned requirements. To maximize clinical usefulness and acceptance, the involvement of different stakeholders (e.g., therapists, patients, engineers and physicians) is essential for the development of rehabilitation devices [81–83]. We thus followed a clinical-driven and human-centered approach with four phases: i) Understand the context of use; ii) Specify the clinical-driven requirements; iii) Develop the solution; and iv) Evaluate against requirements. Results from the steps i) and ii) were reported in [55, 84], and intermediate development steps from iii) in [55, 85, 86]. Here, we present the final robotic system and the final clinical usability evaluation with therapists and stroke patients. We followed a mixed-method approach for the usability evaluations. We combined quantitative methods, i.e., standardized questionnaires with scales and performance-related measures, with qualitative methods, i.e., semi-structured interviews that balance structured queries and personalized dialogues, to obtain a holistic assessment [83, 87].

The rest of the paper is structured as follows: First, we present the development of the novel haptic upper-limb rehabilitation system, addressing the limitations of current robotic rehabilitation devices. This also includes a whole-hand haptic rendering approach, two virtual rehabilitation exercises, as well as a graphical user interface (GUI) for therapists. Then, we present the experimental procedure and the results of a mixed-method clinical usability study with 14 participants (seven therapists and seven sub-acute stroke patients) and discuss our findings.

## Methods

### Clinical-driven requirements

To understand the context of use and to establish the requirements following a human-centered approach, we first spent several days in the Department of Neurology, University Hospital Bern, Switzerland, shadowing clinicians and therapists. We closely followed them during their daily work, taking field notes and asking questions, allowing us to obtain an in-depth understanding of their working environment, procedures, involved people, and interactions [88]. We also performed literature research on previous device developments and studies reporting device requirements studies, e.g., [89–92].

To narrow down the list of requirements, we carried out a 35-question online survey with 33 neurorehabilitation experts (four physicians, one nurse, two speech therapists, one neuropsychologist, six occupational therapists, and 19 physiotherapists, with two thirds of the participants having five or more years of experience) from the University Hospital Bern and Reha Rheinfelden, Switzerland. The detailed results of this survey were reported in [84].

The resulting initial principal system requirements were:

- Collective movement of the index to little finger with a large functional range of motion (RoM) that allows grasp training as well as the training of active finger extension (i.e., full extension required).
- Independent thumb movement, including thumb opposition allowing different grasps such as medium wrap (i.e., cylindrical grasp with contribution of palm), precision disk (i.e., fingertip prehension) and lateral pinch [93, 94].
- Whole-arm movements with a sufficient RoM to perform reach and grasp movements.
- Fast setup (below 5 min), even for patients who cannot fully extend their fingers due to spasticity or hypertonicity.
- Suitable for a wide variety of hand sizes while avoiding many or complicated adjustments.
- Rehabilitation games to train reaching and grasping movements with high-fidelity haptic rendering to provide meaningful sensory information.
- The games’ difficulty must be adjustable to individual patients’ abilities.
- Patient’s movements can be assisted by manually adjustable robotic forces/torques.
- Graphical therapist interface to control the robot and games, e.g., start, stop, and perform adjustments.

Once we started the prototyping phase, we continued seeking feedback on intermediate prototypes from therapists at the University Hospital Bern, Switzerland, in co-creation sessions. In the early stages of the development, we facilitated the conversations by using various 3D-printed prototypes as well as an Omega.3 device (Force Dimension, Switzerland) for the exercises and haptic rendering development.

### Haptic device development

**Fig. 1:**
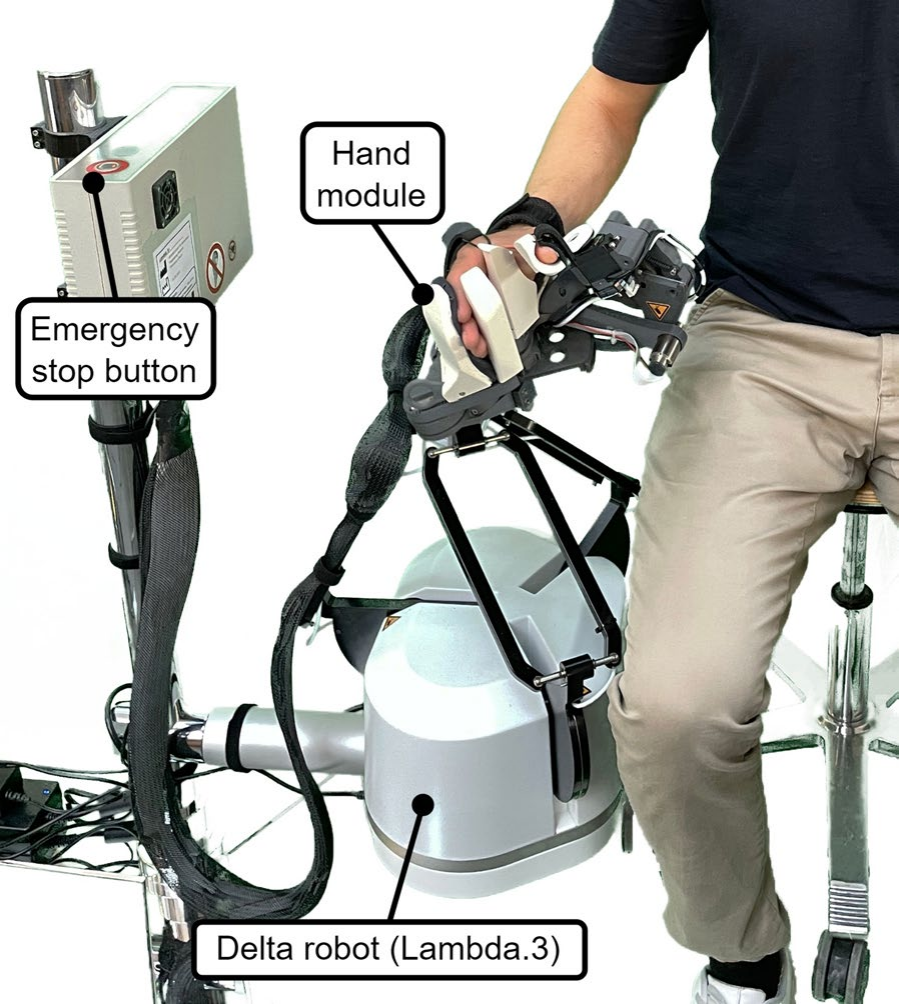
The Lambda.3+ haptic device consists of a delta robot (Lambda.3) and a custom hand module with hand-size-specific exchangeable handles. The user is ready to grasp a virtual object with extended fingers and abducted thumb

**Fig. 2:**
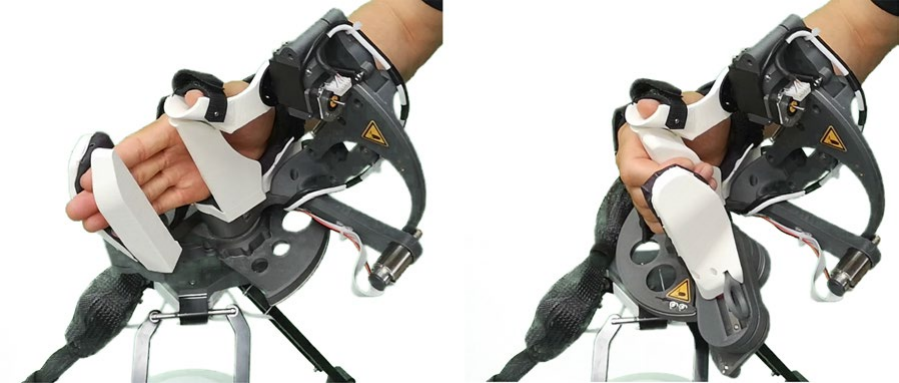
Collective finger flexion and extension movements

**Fig. 3:**
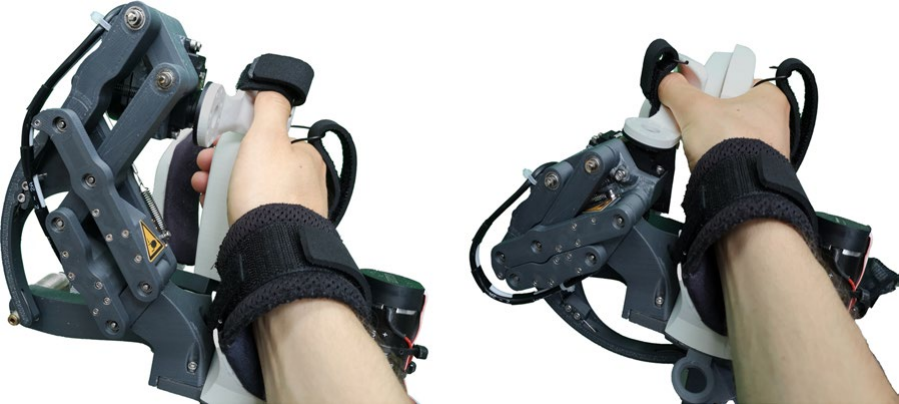
Thumb circumduction movements, enabled by the remote center of motion mechanism

Driven by the established requirements, we designed a novel six-DoF rehabilitation haptic device that consists of a custom-made hand module with three DoF, which allows grasping training, mounted on a delta robotic base (Lambda.3, Force Dimension, Switzerland) with three translational DoF to train reaching movements (Fig. 1). Following, we describe the developments of our modular device that allows: (i) collective finger flexion/extension (Fig. 2), (ii) thumb movements (Fig. 3), and (iii) hand translations.

#### Hand module: collective finger flexion/extension

The design and performance evaluation of the Palmar RehabilitatIon DEvice (PRIDE) for collective finger (i.e., index finger to little finger) flexion/extension movements is described in detail in [55]. We provide here a summary for completeness.

To meet our objective of a short setup, we aimed to minimize the number of adjustable parts. This was also highly encouraged by the therapists in the co-creation sessions. It also became apparent that the hand module should be compact and cylindrically shaped during setup to enable patients with limited finger mobility to slide their hands onto it with flexed fingers. Therefore, a RoM ranging from fully extended fingers to flexed fingers for the setup was required. In consultation with the therapists, we found a finger flexion of 165$^\circ$ (180$^\circ$ for an earlier version of PRIDE), measured as the orientation of the distal segment of the finger with respect to the metacarpal bones, to be adequate. To achieve this, we first categorized the hand sizes given in anthropometric databases [95–98] into four distinct sizes, which we called *S*, *SM*, *ML*, and *L* (from small to large). The *S* corresponds to the 5th percentile of women’s and *L* to the 95th percentile of men’s hand sizes. The intermediate sizes were equally spaced between the smallest and largest sizes. Following, we engineered a mechanism where we used a combination of linkages, pulleys and cables, as well as exchangeable handles (i.e., the part that supports the palm of the hand) to position differently-sized hands. The mechanical design parameters were synthesized an optimization approach.

**Fig. 4:**
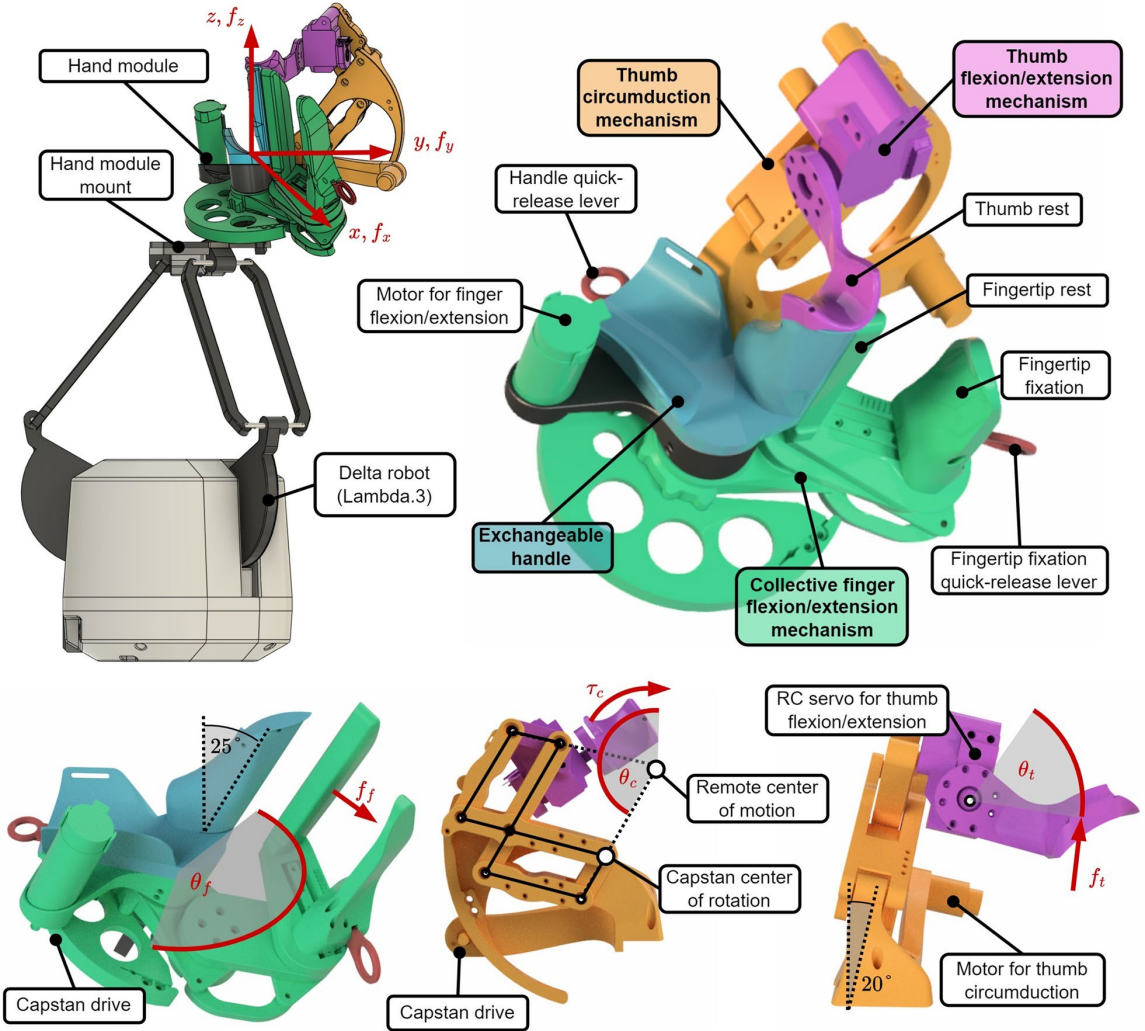
CAD model of the device. Top left: The two main parts are the robotic delta base for translational movements and a custom-made hand module for grasping. Top right: Hand module with exchangeable handle that enables collective finger flexion/extension, thumb circumduction, and thumb flexion/extension. Bottom left: Finger flexion/extension mechanism and inclined handle. Bottom center: Thumb circumduction mechanism. Bottom right: Thumb flexion/extension mechanism

**Fig. 5:**
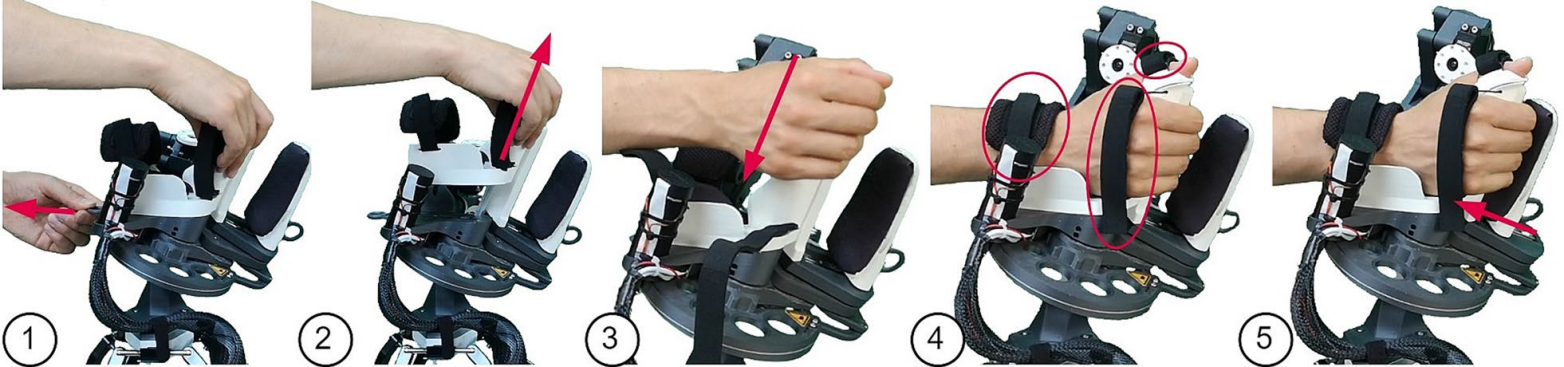
Setup sequence: (1) The handle must be unlocked by pulling the lever on the back (here left side) of the hand module. (2) The handle is being removed by vertically sliding it off the hand module. (3) Once the new handle is installed, the patient’s hand can be slid onto the device without the need for finger extension. (4) The wrist, thumb and metacarpals straps are tightened with hook and loop attachments. (5) The fingertip support is tightened by sliding it towards the fingers and held in place through a ratchet mechanism

The resulting design with minimal mechanical backlash and high transparency—characteristics that are advantageous for haptic rendering—is depicted by the CAD renderings in Fig. 4. To swap the four size-specific handles, they can easily be unlocked and locked with a quick-release lever on the back of the hand module (see Fig. 4). The hand is attached to the handle using two straps: One at the wrist and one over the metacarpal bones. The fingertips are kept in contact with the fingertip support through a custom-made padded fixation on the dorsal side that comfortably presses the fingers against it (Figs. 4 and 5). The fingertip fixation features a ratchet mechanism and can be released by a lever as well. Note that the fingertip support, as well as the handle, are tilted in the coronal plane by 25$^\circ$ as this results in natural cylindrical grasp [99]. The hand module is mostly 3D printed from polylactic acid (PLA) and reinforced with metal parts as needed. The mechanism is actuated through a Capstan drive by an electric motor with an integrated encoder (RE30 with MR Encoder, 1000 PPR, Maxon Motor AG, Switzerland).

#### Hand module: thumb circumduction and flexion/extension

In the co-creation sessions with the therapists, we found that the majority of thumb movements required for the most relevant grasps seem to be achievable by a combination of thumb circumduction and flexion/extension. Despite the complexity of the thumb kinematics [100–102]), the thumb tip can be considered to move on the surface of a cone that originates in the carpometacarpal (CMC) joint during circumduction [103]. We also assumed that only a relatively small flexion/extension RoM is required for the majority of grasps and thus further simplified the thumb movements by approximating the thumb tip path during flexion/extension as a circular arc segment. Initially designed for a RoM of 0°–50°, we limited the RoM further to 30°–50° in software.

Based on this knowledge and assumptions, we developed a two-DoF mechanism to extend the aforementioned PRIDE hand module. We presented the development in detail in [85]. Here, we again only provide a summary for completeness. The movements of the thumb tip support are achieved through a serial kinematic chain. The first revolute joint is realized as a parallelogram-based remote center of motion (RCM) mechanism to avoid collisions with the user’s hand (Fig. 4, bottom center). The axis of rotation of this RCM lies in the coronal plane and goes through the CMC joint with a negative inclination of 20° with respect to the longitudinal axis of the hand. The second revolute joint is located in proximity to the thumb’s MCP joint and enables thumb flexion/extension. To eliminate the need for further adjustments, the exchangeable handles of PRIDE were redesigned such that the vertical (i.e., in radial direction) position of the thumb tip aligns across all hand sizes. To do so, we assumed that the hand breadth scales proportionally to the hand length and added a corresponding offset for the vertical hand position. The design parameters of the thumb mechanism were again synthesized using an optimization approach.

The final thumb mechanism is depicted in Fig. 3, showcasing the circumduction RoM. The mechanism also mostly consists of PLA parts with a few metal parts (e.g., stainless steel axles). The RCM circumduction is actuated through a capstan drive by an electric motor with an integrated encoder (DCX22S with Enc EASY 16, 1000 PPR, Maxon Motor AG, Switzerland). To avoid a high continuous motor load, the RCM mechanism is fitted with a spring that compensates the weight of the moving parts. A geared RC servo (D625MW, Hitec RCD, USA) with an encoder (AMT10E, 5120 PPR, CUI Devices, USA) was used for flexion/extension. The thumb tip is fixated to the thumb tip support with a hook and loop strap (Fig. 5).

#### Delta robot (Lambda.3): hand translation

To enable reaching movements, we installed the hand module on a vertically oriented Lambda.3 device (Force Dimension, Switzerland) with three translational DoF (Fig. 1). The Lambda.3 is a haptic device with parallel delta kinematics and highly backdrivable capstan drive actuation for all three axes. The entire assembly was mounted on a stand with lockable wheels for easy transportation.

The hand module is mounted at an angle of 15° in the sagittal hand plane to  account for the slight wrist extension that naturally occurs during grasping. The mass of the hand module (1700 g) is spring-compensated in the vertical direction by the Lambda.3 device to reduce continuous motor load during operation. We manufactured the hand module in a right-sided and a left-sided version, whereas the same Lambda.3 robot was used for all participants.

The resulting rehabilitation robot (see Fig. 1) has a total of six DoF and falls into the group of end-effector-style devices [16]. The workspace and force/torque specifications of the complete rehabilitation robot are summarized in Table 1.

**Table 1:** Device specifications

Axis	Range of motion	Force/ torque
XY ($x_m, y_m$)	Ø 240 mm	20 N
Z ($z_m$)	170 mm	20 N
Finger flexion/extension ($\theta _{f,m}$)	0$^{\circ }$ - 165$^{\circ }$	>30 N$^*$
Thumb circumduction ($\theta _{c,m}$)	0$^{\circ }$ - 80$^{\circ }$	0.4 Nm
Thumb flexion/extension ($$\theta _{t,m}$$)	0$$^{\circ }$$ - 50$$^{\circ }$$ $$^{**}$$	14 N

*For fully extended fingers. Varies due to non-constant mechanical advantage. **Limited in software to approx. 30°–50°

### Haptic rendering and control

#### Control hardware

While the Lambda.3 robotic base comes with integrated control electronics, we used an additional control box that was custom-made by Force Dimension to drive the three motors of the hand module. Both controllers were connected via USB to a host PC and both were equipped with an independent hardware watchdog timer. Triggering a watchdog timer shuts all motors off immediately and shortcuts the motor drivers, resulting in electromagnetic viscosity that prevents the device from collapsing too quickly. The control box of the hand module was also equipped with an emergency stop button (see Fig 1, button with red marking on top of the control box) that, when pressed by the responsible therapist, has the same effect as the watchdog.

#### Physics engine-based whole-hand haptic rendering

**Fig. 6:**
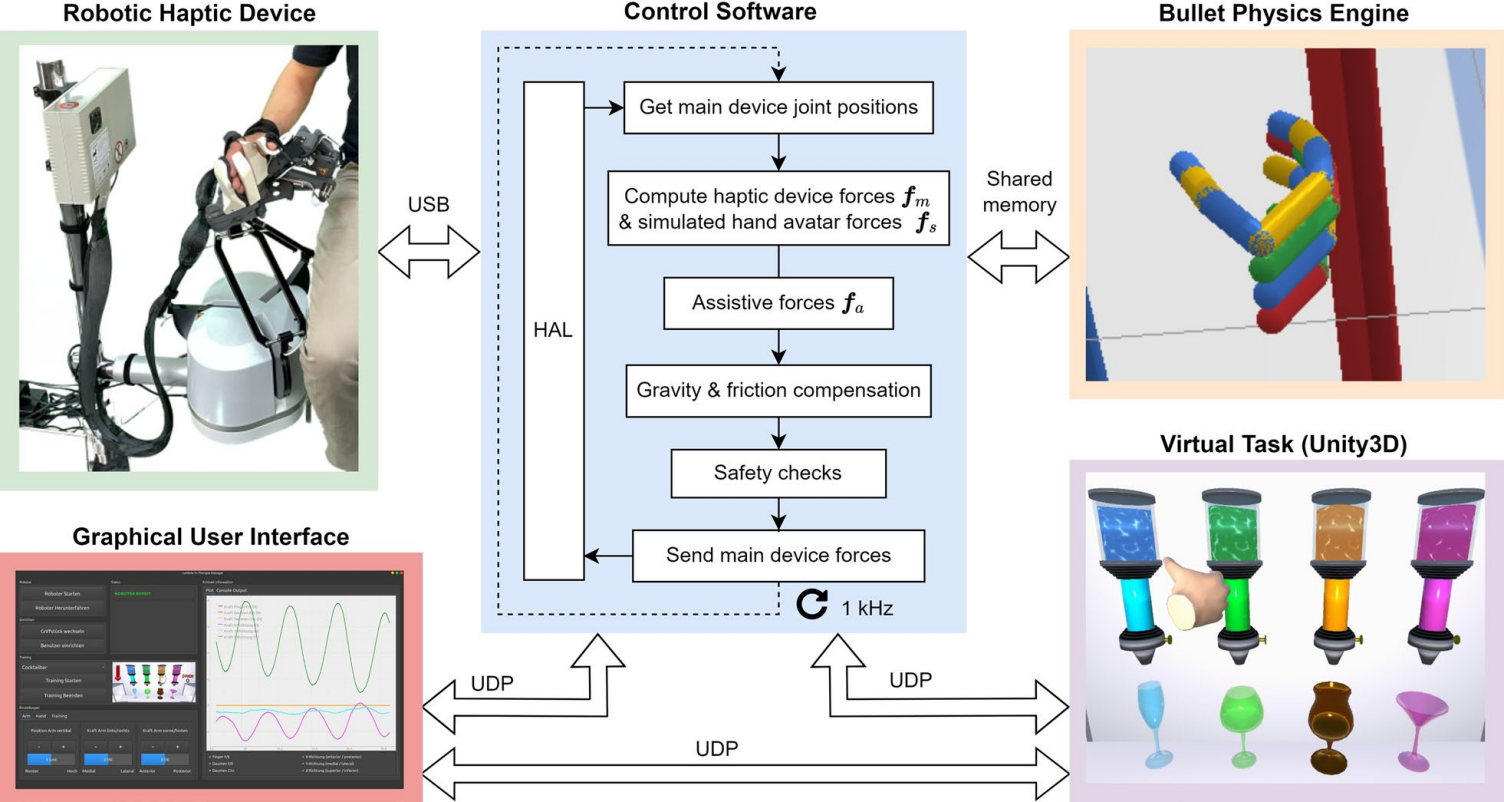
Overview of the software architecture. Except for the Bullet physics engine server which communicates through shared memory with the control software, the different software modules communicate using UDP. The main control software computes the haptic forces at a rate of 1 kHz and interacts with the robotic devices through a hardware abstraction layer (HAL) using a USB connection

We developed a haptic rendering method based on the open-source physics engine *Bullet* to render whole-hand interactions between the patient’s hand (virtually represented by a hand avatar; Fig. 6) and virtual objects. Here, we provide a simplified overview and refer the reader to [86] for more details. Our approach essentially consists of a bilateral proportional-derivative (PD) control law [104] that couples a main device (i.e., our haptic device, denoted in following with index ) and a simulated hand avatar, denoted with index .

The generalized device coordinates $\varvec{q}_m = [x_m, y_m, z_m, \theta _{f,m}, \theta _{c,m}, \theta _{t,m}]^{\hspace{-0.83328pt}{\textsf{T}}}$ and the forces/torques $\varvec{f}_m = [f_{x,m}, f_{y,m}, f_{z,m}, f_{f,m}, \tau _{c,m}, f_{t,m}]^{\hspace{-0.83328pt}{\textsf{T}}}$ are indicated in Fig. 4 and Table 1. The simulated hand avatar was modeled as a multi-body object using capsule colliders (Fig. 6), whereas its DoFs correspond to the DoFs of our device. Its generalized coordinates $\varvec{q}_s$ and its forces $\varvec{f}_s$ are, thus, defined analogously.

The virtual forces applied in the physics engine to the simulated hand avatar ($\varvec{f}_s$) are given in Eq. (1), and the haptic rendering forces sent to the main haptic device ($\varvec{f}_m$) are given in Eq. (2). The diagonal matrices $\textbf{K}_{p,m}$, $\textbf{K}_{p,s}$ and $\textbf{K}_{d,m}$, $\textbf{K}_{d,s}$ represent proportional and damping gains, respectively. The matrix $\tilde{\textbf{M}}$ denotes the mass matrix of the simulated hand avatar (diagonal elements only, see [86]), and $\varvec{h}$ is the lumped term for velocity and gravity-dependent forces. The diagonal matrix $\mathbf {\Phi }$ in Eq. 2 is an adaptive damping gain that stabilizes the patient’s hand, fingers or thumb upon impact with a virtual object while only slightly degrading the sensations of hand-object impacts or hampering movements in free space. The term $\varvec{f}_e$ in Eq. 2 is a vector of additional exercise-specific forces (see Section "Virtual training tasks") and $\varvec{f}_a$ represents adjustable assistive forces (see Section "Therapist interface and device operation").

1 $$\begin{aligned} \varvec{f}_s= & {} \tilde{\textbf{M}} \left( \textbf{K}_{p,s}(\varvec{q}_m - \varvec{q}_s) + \textbf{K}_{d,s}(\dot{\varvec{q}}_{m} - \dot{\varvec{q}}_{s}) \right) + \varvec{h} \end{aligned}$$

2 $$\begin{aligned} \varvec{f}_m= & {} \textbf{K}_{p,m}(\varvec{q}_s - \varvec{q}_m) - \mathbf {\Phi } \textbf{K}_{d,m}\dot{\varvec{q}}_{m} + \varvec{f}_{e} + \varvec{f}_a \end{aligned}$$

#### Software implementation

In Fig. 6, the different software modules including the therapeutic games described in Section "Virtual training tasks" and the therapist interface in Section "Therapist interface and device operation" are depicted. The control software—responsible for low-level control, haptic rendering, and safety checks—was written in C++ and communicates through a hardware abstraction layer (HAL) with the hardware described above. In addition to the hardware safety features, the control software also continuously monitors the device’s velocity and the motor encoders, and disables all forces in case of an operational anomaly. The mechanical gravity compensations of the Lambda.3 and hand module are complemented by fine-tuned gravity and friction compensation in software. A scaling factor of six between the translational movements of the haptic device and the hand avatar movements was introduced to adjust the virtual translational workspace in the games to the available workspace of the device. The finger and thumb movements were mapped 1:1 between the patients’ hands and the simulated hand avatar to provide congruent proprioception-visual information during grasping.

The control software update loop runs at a rate of 1 kHz. All software modules ran simultaneously on the host computer (AMD Ryzen 7 PRO 5850U, 32GB, with Ubuntu 22.04 and a low-latency kernel). Except for the Bullet physics engine, where shared memory was used, the communication between the different software modules was performed through socket networking using the User Datagram Protocol (UDP).

### Virtual training tasks

#### Overview

We developed two gamified training tasks to complement our device, where we targeted reaching, grasping and releasing objects. We aimed to design tasks that benefit from meaningful haptic feedback, i.e., easily interpretable sensory information that contributes to controlling the movements and facilitates task completion for simultaneous motor and sensory training. While the first game (cocktail bar game) was inspired by therapy tasks and endorsed by therapists, we organized brainstorming sessions with two therapists for the development of the second game (slingshot game) [105]. Both games were developed in an iterative process through informal feedback from repeated intermediate testing by therapists. The graphical elements and game logic were developed in Unity3D (Unity Technologies, USA) in C#, while haptics-related elements were implemented as part of the control software (see Section "Haptic rendering and control").

Although our robotic device is capable of rendering forces in all DoFs, we decided to block the vertical axis (*z*-axis) with a PD controller for three reasons. First, during informal testing with therapists in the co-creation sessions, we found that, likely due to the 2D screen, performing straight and targeted 3D grasping movements seemed challenging—a finding also supported by literature [106]. In particular, vertical and horizontal (i.e., forward/backward) movements could be indistinguishable depending on the perspective on the 2D screen. Thus, we agreed with the therapists that the games were challenging enough with a blocked *z*-axis, and that we did not want to add another dimension of difficulty and source of ambiguity. Second, this allowed us to adjust the height of the device during training. Finally, we prevented any adjustment or calibration that might have been required with dynamic arm-weight support [107].

#### Cocktail bar game

**Fig. 7:**
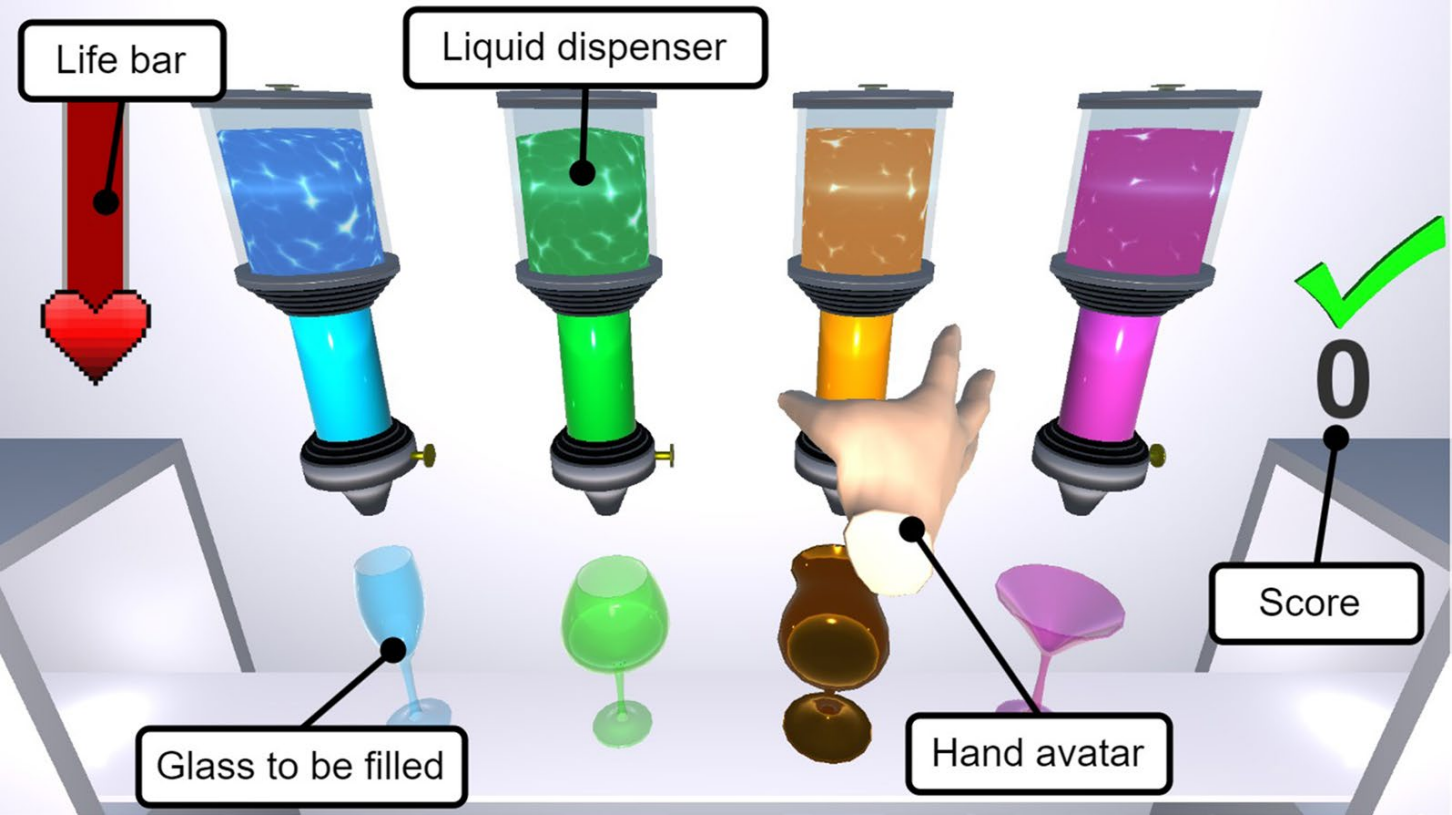
Cocktail bar game: The patient has to grasp liquid dispensers with different haptic characteristics and skillfully squeeze them to pour liquid into the glasses

The first game was designed to promote proximal and distal movements with a special focus on fine distal movements and force dosage. In the cocktail bar game (Fig. 7), the patients are presented with four liquid dispensers, each containing a differently colored liquid with a distinct viscosity. The user’s hand is represented by a hand avatar, which reflects the user’s real hand movements. Below each dispenser, a glass appears that must be filled with the liquid. For this, the user has to move the hand avatar in front of the dispenser, open the hand, bring the thumb in opposition, approach the dispenser, close the hand until the fingers “touch” it, and then squeeze to pour liquid. If the hand is not properly opened before approaching the dispenser, the dorsal side of the fingers collide with it and the user will feel that the fingers are pushed into a flexed position, analogous to a real-life scenario. Thus, the opening of the hand before grasping is necessary, meeting the therapists’ request for the integration of active finger extension in the training of functional movements.

Importantly, the liquid and dispenser physical characteristics (i.e., viscosity and stiffness) are reflected in the haptic rendering of the interaction between the patient’s hand and the dispenser. Whenever the dispenser is squeezed too hard, the liquid spills and sputters. Whenever liquid is spilled beside any of the glasses, the user is penalized, i.e., the life bar located on the top left of the screen decreases (Fig. 7). Hence, the challenge is to skillfully squeeze the liquid dispenser based on the visuo-haptic feedback. As soon as a glass is full, a green check mark appears, the glass disappears, and the counter of successful glasses on the right of the screen increments. In a first step, each one of the four glasses needs to be filled once. Afterward, the glasses appear randomly upon completion of the preceding one. The difficulty could be varied by scaling the required forces to pour liquid from the dispensers (i.e., facilitating movements for patients with low residual grasping force) and by adjusting the sensitivity of the dispensers (i.e., how quickly they start to sputter and spill liquid).

For the haptic rendering of the hand-dispenser interaction, a virtual wall (i.e., virtual spring-damper system with gains $k_{p,e}$ and $k_{d,e}$, respectively) to the finger flexion/extension movement is superimposed to the whole-hand interaction forces from the simulated collisions in the physics engine (force $f_{e}$ in Eq. 2, Section "Haptic rendering and control"). The gains $k_{p,e}$ and $k_{d,e}$ can be modified based on the desired impedance characteristics of the corresponding liquid dispenser. The virtual wall is activated during the grasping of a dispenser shortly before the fingertips hit the cylindrical collider representing the liquid dispenser (at 90$^\circ$ finger flexion). Since the fingertips are occluded—i.e., behind the dispenser—in this position, the virtual wall is perceived as the interaction between the patient’s hand and the dispenser. Although this approach does not perfectly model a deformable liquid dispenser, it works very well in practice, requires only minimal additional computational cost, and simplifies the development compared to actually utilizing deformable meshes or modifying the properties of the liquid dispenser colliders in an online manner. The exercise-specific finger flexion/extension force $f_{e,f}$ is calculated as a virtual wall using Eq. (3) with $\Delta \theta _{f,m}$ denoting the penetration into the virtual wall. The other components of the vector $\varvec{f}_e$ remain zero.

3 $$\begin{aligned} f_{e,f} = {\left\{ \begin{array}{ll} k_{p,e} \Delta \theta _{f,m} + k_{d,e} \dot{\theta }_{f,m}, &{}\quad \Delta \theta _{f,m} > 0\\ 0, &{}\quad \text {otherwise.} \end{array}\right. } \end{aligned}$$

#### Slingshot game

**Fig. 8:**
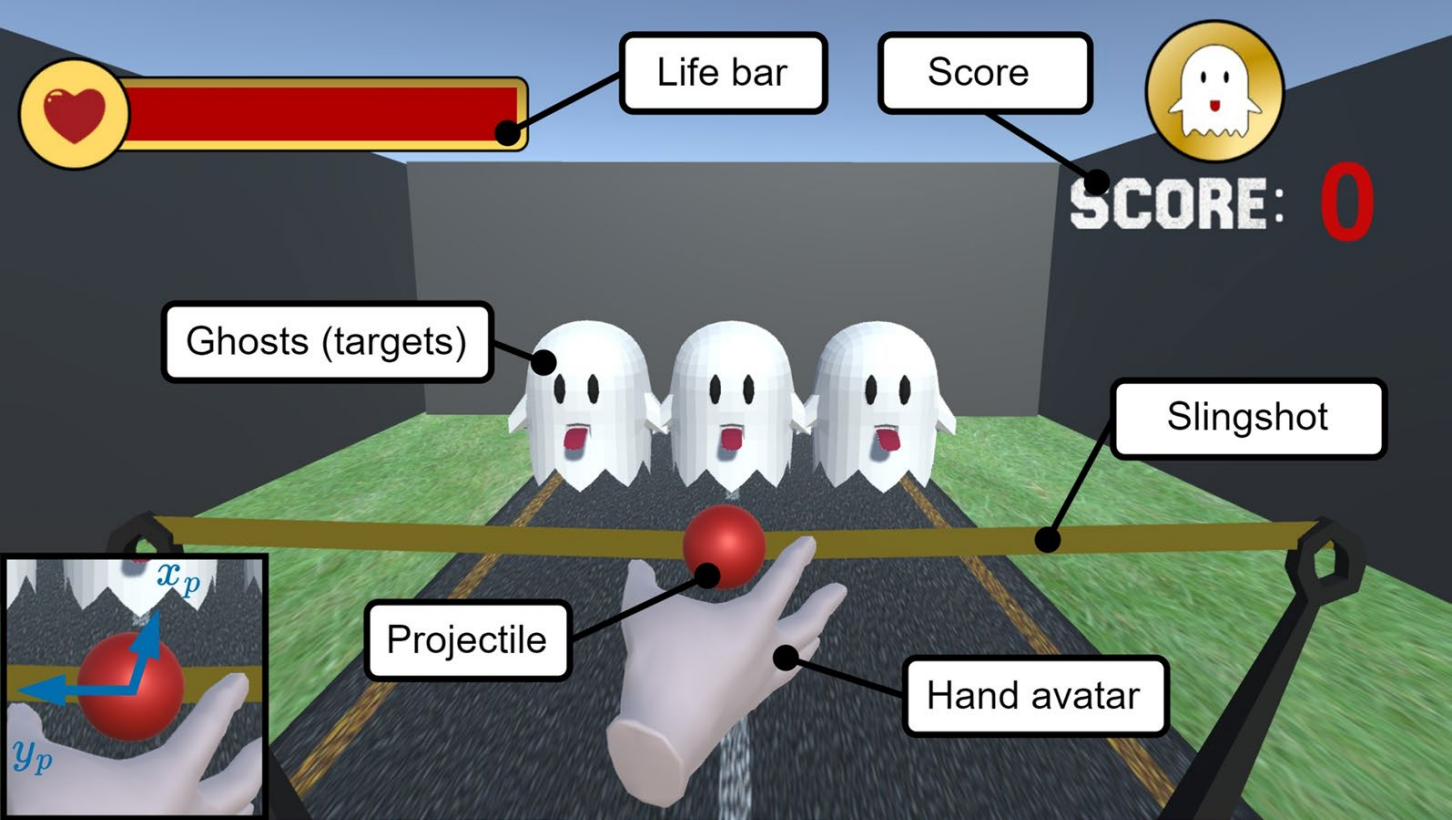
Slingshot game: The projectile (close-up in the bottom left corner) must be grasped and pulled back and released to shoot with the slingshot. The goal is to hit the ghosts

In this game, the therapeutic objective is to train fine proximal movements while still requiring distal movements during grasping. The goal is to hit ghosts that appear on the screen using a simulated slingshot (Fig. 8). For this, the user first reaches out to grasp a projectile (i.e., the red ball) at the screen’s center. Grasping requires opening the hand, opposing the thumb, and closing the fingers around the projectile. Once the user thinks that the projectile is correctly grasped, the slingshot can be tensioned by pulling back and released by opening the hand. To aim, the arm can be moved left and right, with the projectile’s expected trajectory shown on the screen.

The game increases difficulty as the patient plays. In the first phase, three stationary ghosts are presented. Once the three initial ghosts are shot, three new ghosts appear that move continuously from left to right and back. Finally, ghosts randomly appear and move towards the user. In this phase, whenever the user misses a ghost and the ghost passes the slingshot, the life bar, depicted on the top left of the screen (Fig. 8), decreases. The difficulty of the game can be further adjusted by scaling the user’s input forces, i.e., the necessary forces for grasping and pulling back the slingshot can be reduced to facilitate the task.

To simulate a slingshot, a virtual spring-damper system is temporarily attached to the projectile with coordinates $x_p$ and $y_p$ (Fig. 8). The dynamics of the projectile are governed by the force $\varvec{f}_p$ from Eq. (4) and the hand-projectile interactions. Thereby, the diagonal matrices $\textbf{K}_{p,p}$ and $\textbf{K}_{d,p}$ denote the stiffness and damping. To facilitate grasping, the stiffness of the slingshot in frontal direction (away from the user) is increased by the scalar factor $\gamma \in [1, \hat{\gamma }]$, which is computed according to Eq. (5). The peak value $\hat{\gamma }$ was chosen $\hat{\gamma } = 3$ in our experiments. This allows the user to push the hand against the slingshot to get a good grasp before pulling back. By either opening the hand or reducing the grasping pressure, the slingshot forces on the projectile will overcome the simulated friction between the hand avatar and the projectile and catapult it in the direction where the user aimed. The virtual spring-damper system is detached from the projectile when a release is detected, i.e., when the projectile surpasses a velocity threshold.

4 $$\begin{aligned} \varvec{f}_p= & {} \gamma \left( \textbf{K}_{p,p} \begin{bmatrix} x_p\\ y_p \end{bmatrix} + \textbf{K}_{d,p} \begin{bmatrix} {\dot{x}}_p\\ {\dot{y}}_p \end{bmatrix} \right) \end{aligned}$$

5 $$\begin{aligned} \gamma= & {} {\left\{ \begin{array}{ll} \hat{\gamma }\left( 1 - \frac{2}{\pi } \big | \text {atan2}(y_p, -x_p)\big | \right) , &{}\quad x_p < 0\\ 1, &{}\quad \text {otherwise.} \end{array}\right. } \end{aligned}$$

### Therapist interface and device operation

**Fig. 9:**
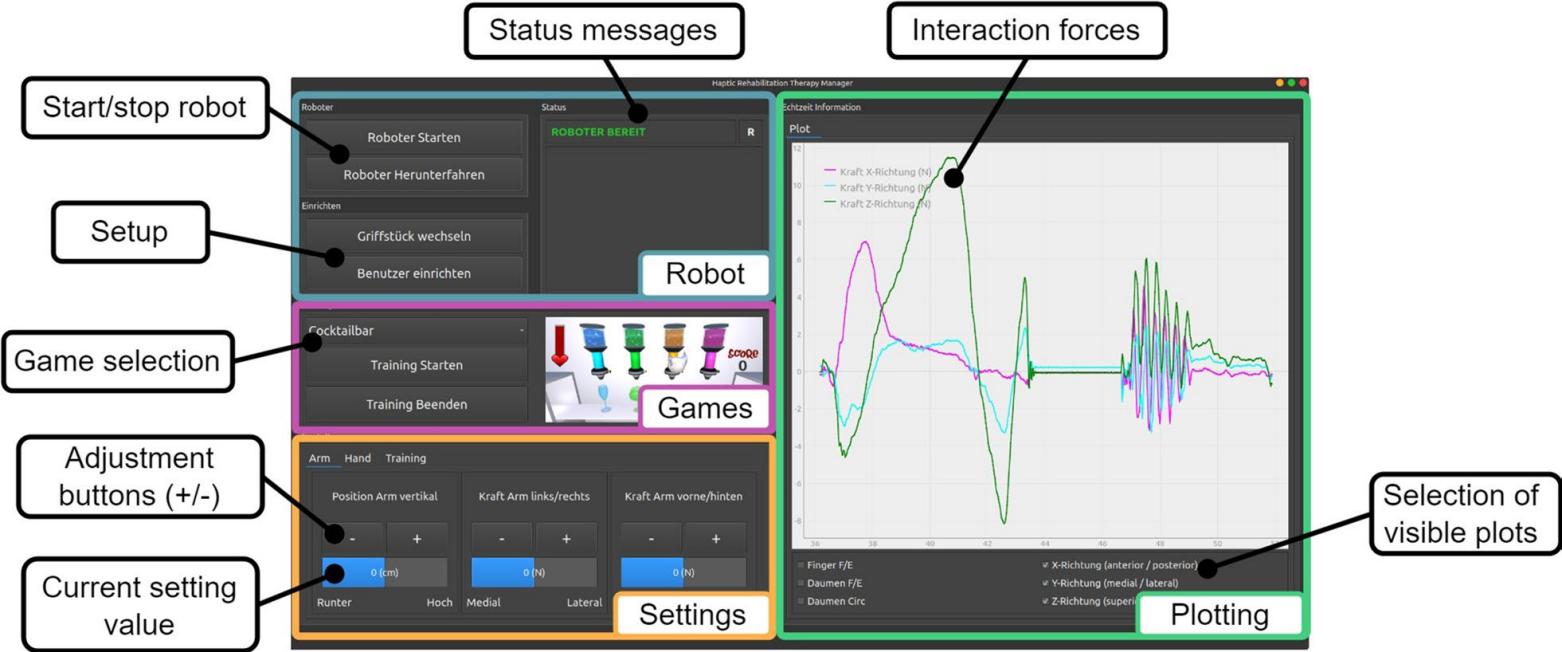
Graphical User Interface (GUI) for the therapists. In the section *Robot*, the robot can be started, prepared, and stopped. In *Games*, a game can be selected and started or stopped. The section *Settings* allows to make game-specific difficulty adjustments as well as adjustments of the device height and assistive forces. In the *Plotting* area, the estimated patient-device interaction forces are plotted

The graphical user interface (GUI) is the main interaction channel between the therapist and the system. When it comes to the design, we aimed for a logical flow, where therapists start at the top left and successively work their way down to the bottom and where elements with related functionalities are grouped together. Our goal was to provide a minimum set of functionalities that were necessary to operate the device without increasing the overall system complexity. The resulting GUI (see Fig. 9) was developed in Python using Qt (The Qt Company, Finland) in German, the local language at the study hospital.

The GUI is divided into four distinct sections: (i) In the *Robot* section, the robotic device can be started, stopped and prepared for swapping handles or setting up the patient. The current status of the robot (e.g., disconnected, ready, busy, error messages, etc.) is displayed. (ii) The *Game* section lets the therapist select, start, and quit a game. (iii) In the *Settings* section at the bottom left of the GUI, adjustments to the height of the robot, the assisting forces, and game difficulty can be set in three separate tabs. (iv) In the *Plotting* area on the right side, a real-time plot of the estimated forces (using the motor current) that the patient applies is shown. The plotting was not an explicit requirement from therapists, but since they were curious about the magnitude of the applied forces during the co-creation sessions, we decided to add it. The desired DoFs for which the forces should be plotted can be selected using tick boxes.

The sequence to initiate a training game is as follows. After starting the device with the start button in the *Robot* section, the robot moves close to the patient into a predefined position. The therapist can then install the correctly sized handle for the specific patient prior to the patient setup by first pressing a button in the *Robot* section. By the press of this button the hand module moves into an adequate configuration to make the handle easily accessible, i.e., thumb support at full circumduction and extension, finger support at moderate flexion). During the handle swapping, the GUI provides visual instructions to the therapist in a pop-up window. Once the correct handle size is mounted, the therapist presses the last button in the *Robot* section and the hand module automatically moves to a fully closed configuration for easy installation of the patient’s hand in the device. The therapist is then assisted in donning the patient by instructions displayed in a pop-up window. The complete setup sequence of changing the handle and installing a patient is depicted in Fig. 5. Once the patient is installed, the therapist can select the game in a drop-down menu and start the therapy.

To adjust the training to the individual patient’s needs, the *Settings* section offers several adjustments. This section consists of the tabs *Arm*, *Hand*, and *Training*. In the first two tabs, the height of the robot can be adjusted, and for each of the remaining five DoFs, an assistive force (see Section"Haptic rendering and control") can be adjusted in either direction. The height adjustment is performed with a minimum-jerk trajectory to be perceived as smooth by the patient [108]. The assisting force magnitude can be changed using plus / minus buttons and displayed on a linear gauge. The *Training* tab lets therapists adjust the game difficulty as described in the Section "Virtual training tasks". Importantly, the use of the settings is not required, and therapists are free to use them according to their preferences and patients’ needs. All the settings can be adjusted online while the patient is playing the game.

### Usability study

#### Participants

We performed our usability study with seven therapists and seven stroke patients. The study was approved by the cantonal ethics committee (BASEC number 2018-01179) and Swissmedic (case number 10000432) and complied with the Declaration of Helsinki.

To obtain insights from different points of view, we included three physiotherapists (PT) and four occupational therapists (OT). The therapists were recruited through word of mouth by their respective group heads at the University Neurorehabilitation, Inselgruppe, Inselspital Bern and Spital Riggisberg, Inselgruppe, Riggisberg, Switzerland. There were no inclusion criteria for the therapists, except that they had to be fit for work.

The patients were recruited through their respective therapists with a printed flyer that explained the experiment in simple words. All participating patients were actively undergoing conventional rehabilitation at the time of the study. The patient inclusion and exclusion criteria were defined relatively loosely as the responsibility of choosing suitable patients was given to the respective therapists. The inclusion criteria were: Mildly to moderately impaired adult stroke patients with residual upper-limb motor capabilities and none to moderate spasticity. Exclusion criteria were: Shoulder instabilities or other conditions that would increase the risk of injury when using a robotic device and notable cognitive or visuo-spatial deficits that would likely influence the experiment outcome (e.g., neglect, aphasia, confabulation). These criteria were not evaluated by means of standardized assessments. Instead, we provided them as guidelines for the therapists who ultimately decided if a patient was suited for participation based on their daily experience in the conventional therapy program.

#### Study protocol

The experimental setup consisted of the robotic device, a laptop with a 14” screen and a computer mouse, an external 24” monitor, and chairs for the therapist and patient. The laptop, computer mouse, and external monitor were placed on a table, and the therapist and the patient were sitting in front of it next to each other. The games were displayed on the external monitor in front of the patient, while the GUI for the therapists was displayed on the laptop screen.

**Fig. 10:**
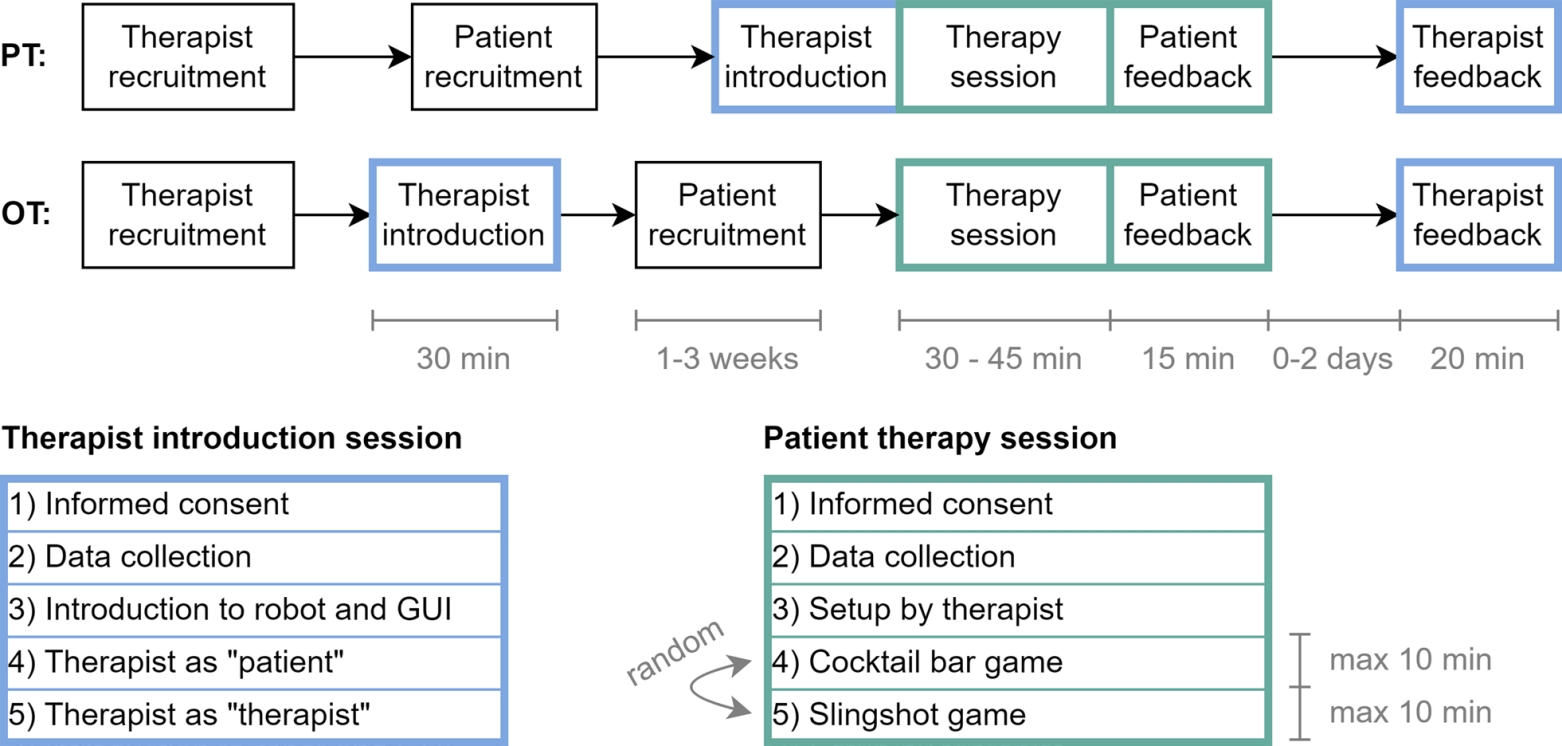
Usability study protocol. The study protocol was slightly different for the physiotherapists (PT) and occupational therapists (OT) for organizational reasons. Note that the order of the games was randomized and that therapists were allowed to switch between games if desired

The study was divided into two consecutive sessions (see Fig. 10): A first *therapist introduction session* to familiarize the participating therapist with the system and a following *patient therapy session* where the therapist used the robotic system with a patient. Feedback was gathered in separate feedback rounds from therapists and patients after the second session. Both sessions began with the explanation of the study’s goal and the acquisition of informed consent, followed by the collection of data that was not directly related to the system usability (e.g., age, handedness, hand dimensions; see Section "Outcome measures"). Both sessions took place in the therapy facilities of the University Neurorehabilitation, Inselgruppe, Inselspital Bern, and Spital Riggisberg, Inselgruppe, Riggisberg, Switzerland. One of the participating patients was English-speaking. The respective therapist was fluent in English as well. All other experiments were performed in Swiss German. Both sessions were guided by the same lead experimenter (male rehabilitation robotics engineer) and assisted by a second researcher (female neuroscientist with clinical experience). The therapists performed the experiments within the scope of their employment and did not receive any supplementary compensation aside from an ice cream voucher to acknowledge their participation. The patients did not receive any compensation.

The study protocol slightly differed for PT and OT due to organizational reasons. The PTs recruited patients before the therapist introduction session and conducted the therapist introduction session and the patient therapy session on the same day. The head of the PT was well-informed about the device, allowing for an appropriate selection of patients, even without introducing the corresponding PT to the system. The OTs received the introduction first, recruited a suitable patient in the following days, and only then performed the patient therapy session (see Fig. 10).

During the *therapist introduction session*, the participating therapist was first introduced to the robot and GUI by the lead experimenter. For consistency, the system was explained with the aid of a printed quick-start manual (see Additional file 1). Next, the therapist was given the role of a patient—i.e., remaining passive and awaiting instructions, but without simulating any specific pathology—while the system was operated by the lead experimenter. For this, the therapist’s dominant hand was first installed in the device by the experimenter following the instructions on the GUI (see Section "Therapist interface and device operation"). Then, all functionalities were showcased to the therapist in approximately ten minutes. Consecutively, the roles were switched, and the therapist operated the device while the experimenter took the role of the patient for approximately ten minutes. From the beginning, the therapist was encouraged to ask questions.

During the *patient session*, the respective therapist was asked to set up the patient’s affected hand in the system and carry out two virtual rehabilitation exercises—i.e., the cocktail bar and slingshot games—with the patient. The therapist was instructed to use each exercise for a maximum of ten minutes. The exercise order was randomized. The therapist led the session and was free to switch between games as desired or to conclude the session before the full 20 min of gaming time. The therapists could continuously adjust the assistive forces as well as the game difficulty according to their perception of the patient’s specific needs and performance. The maximum game duration was chosen so that the entire experiment was guaranteed to fit into a standard therapy session of 45 min. Therapists operated the system independently; however, they were allowed to consult the provided printed quick-start manual or to ask questions to the lead experimenter if needed.

Feedback on the system usability in the form of standardized questionnaires and semi-structured interviews was gathered from the patients directly after the patient therapy session (see Section "Outcome measures"). Depending on the therapists’ availability, their feedback was collected right after the patient therapy session as well, or at a later time, but with a maximum of two days after the experiment. In this case, the semi-structured interview was performed via a phone call.

#### Outcome measures

##### Main outcomes

First, we measured how long it took the therapists to change the handle of the hand module and how long it took them to install the patient’s hand (including positioning of the robot if necessary) during the patient therapy session. Note that before that, the therapists had only performed the setup of a user once during the therapy introduction session with the experimenter but never with a patient. The handles were pre-installed so that every therapist had to perform a handle change. Moreover, we also assessed the time spent performing each of the two therapy games.

We used the System Usability Scale (SUS) questionnaire to assess the perceived usability from both the therapists and patients [109]. The SUS consists of ten five-point Likert-style items ranging from “Strongly disagree” to “Strongly agree” whereas five items are formulated positively and five negatively. We assessed the therapists’ perceived usability of the GUI (including game adjustments) and the robotic device separately to distinguish any potential usability issues stemming from the human-software interface (GUI) or from the human-hardware interface (robot). We did not add a SUS specifically for the virtual training games because we prioritized the evaluation of tasks commonly undertaken by therapists during therapy sessions (e.g., operating the GUI or interacting with the robot during setup). However, we additionally employed the Perceived Usefulness (PU) questionnaire of the Technology Acceptance Model (TAM) [110] to assess the degree to which therapists believe that using the system would help them accomplish their daily work. It consists of six positively formulated seven-point Likert-style items ranging from “Unlikely” to “Likely” with the option “Not applicable”. We also evaluated the perceived system (robot and game) usability from the point of view of the patients with the SUS questionnaire.

Given the importance of motivation in driving effort and engagement during robotic training for stroke patients [111], we additionally employed the Interest/Enjoyment subscale of the Intrinsic Motivation Inventory (IMI) to measure patients’ motivation. This subscale consists of five positively and two negatively formulated seven-point Likert-style items ranging from “Not at all true” to “Very true” [112].

The complete questionnaires employed can be found in the Additional file 2. All questionnaires were completed by the participants using pen and paper. No writing was required, as choices could simply be marked, allowing patients to use their non-paretic hand even if it was not their usual writing hand. The English-speaking participant received the original English SUS and IMI questions.

To facilitate the interpretation of the results, we normalized the questionnaire scores to a 0–100% range. The SUS and PU scores, as well as the IMI subscale scores per participant, were computed by averaging across all items. We report the central tendency of the questionnaires as median with first and third quartiles due to the low sample number and the ordinal nature of the data.

Qualitative usability data from both therapists and patients was acquired via open-ended questions in semi-structured interviews (see questions in Additional file 2). These questions served to guide the discussion, but the experimenters delved deeper into topics that appeared to be important to each participant. The participants’ answers to the semi-structured interviews were recorded in writing by the experimenters in German or English, respectively, in the case of the English-speaking patient. For the analysis, the participants’ answers were translated to English with DeepL Pro (DeepL SE, Germany) in an initial step and subsequently further refined manually. To identify recurring patterns, opinions, and ideas from these interviews, a thematic analysis was performed by the lead experimenter. This systematic approach involved labeling relevant text segments with codes (i.e., designated descriptive labels), organizing these codes into cohesive themes, and subsequently summarizing and reporting the findings. For a detailed description of the procedure, please consult [113].

##### Secondary outcomes

 We also collected secondary outcome measures that do not directly assess the usability. Apart from demographic information, we noted whether the participants had any previous experience with robotic rehabilitation devices. Additionally, the secondary experimenter took notes of unexpected events (e.g., patient discomfort or technical issues) during the experiments.

We also noted the handle size that was used and measured the participants’ length of the middle finger (from the center of the MCP joint to the tip), the overall hand length (from the distal crease of the wrist joint to the furthest fingertip) and the hand breadth. We then compared the participants’ hand dimensions to the hand dimensions assumed during the design of the hand module to inform the interpretation of potential comfort-related issues. To do so, we first computed the pooled mean and standard deviations of the measurements of male and female hands based on the anthropometric databases that we used for the development of the hand module. The hand length and breadth were directly taken from the measurements of Garrett [96, 97], while the length of the middle finger was computed using the hand length from Garrett and the scaling factor for the middle finger suggested by Buchholz [95]. We then compared these reference values to the combined hand measurements (i.e., from therapists and patients) from our experiments with independent t-tests. The analysis was performed using the SciPy package in Python.

## Results

### Participants

An overview of the therapists’ and patients’ demographic information is presented in Tables 2 and 3, respectively. The median age of the therapists was 37.0 (28.0–57.0) years, with 13.0 (5.0–32.0) years of professional experience. The median age of the patients was 49.0 (45.0$-$54.0) years. Three patients were in the early sub-acute stage, two in the late sub-acute stage and two in the chronic stage according to the classification provided in [114]. Therapists and patients worked in dyads according to their identifiers, e.g., T1 with P1.

Six therapists reported previous experience with robotic devices, including both upper-limb and lower-limb devices, yet none of them had worked with a haptic rehabilitation device before. Three of the patients already used the Armeo^®^Spring in their current rehabilitation program. Although the Armeo^®^Spring is not actuated, it was still perceived as a robotic device by therapists and patients. Patient P6 reported experience with robotics from building virtual reality racing simulators as a hobby.

**Table 2:** Demographic information of the therapists

ID	Age	Sex	Handedness	Profession	Professional experience (years)	Robotic experience	Handle size
T1	37	Female	Right	OT	13	None	SM
T2	25	Female	Right	OT	2	GM, Pablo, Diego	S
T3	62	Female	Right	PT	40	CM	SM
T4	65	Male	Right	PT	37	AS, YG	L
T5	52	Female	Right	PT	27	YG, CM	S
T6	31	Female	Right	OT	7	Diego, GM	S
T7	25	Female	Right	OT	3	AS	S

GM: Gloreha Maestro, Pablo: Tyromotion Pablo^®^, Diego: Tyromotion Diego^®^, YG: YouGrabber, AS: Hocoma Armeo^®^Spring, CM: Motek C-Mill treadmill

**Table 3:** Demographic information of the patients

ID	Age	Sex	Handedness	Paresis	Robotic experience	Handle size	Patient type	Weeks since incident	Lesion
P1	53	Male	Right	Right	AS	SM	Inpatient	21 (late sub-acute)	Ischemic at anterior choroidal artery left
P2	45	Male	Right	Left	AS	SM	Inpatient	21 (late sub-acute)	Intra-cerebral bleeding basal ganglia right
P3	45	Female	Right	Right	None	ML	Inpatient	7 (early sub-acute)	Post-operative ischemia in caudal mesencephalon and pons left lateral
P4	40	Female	Right	Left	None	S	Outpatient	72 (chronic)	Intracerebral basal ganglia hemorrhage
P5	61	Male	Right	Left	AS	ML	Inpatient	28 (chronic)	Ischemic at basilial occlusion, incl. posterior inferior cerebellar occlusion
P6	49	Male	Left	Right	None$^*$	SM	Inpatient	5 (early sub-acute)	Multiple lesions
P7	55	Female	Right	Right	None	S	Inpatient	5 (early sub-acute)	Infarction in superior cerebellar artery

AS: Armeo^®^Spring

*Patient reported experience with haptic racing simulators

### Setup and game play durations

**Fig. 11:**
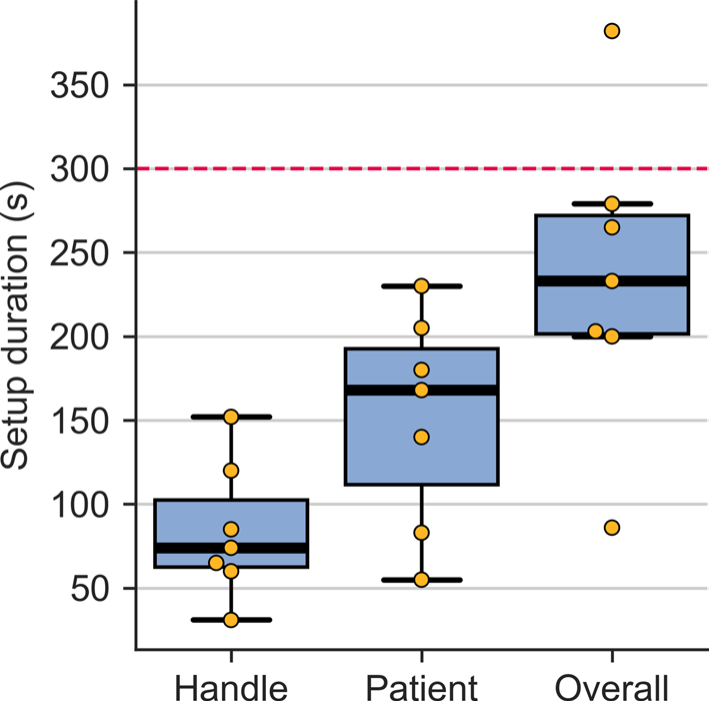
Duration required by therapists for the setup. Handle: Change of the handle; Patient: Duration of positioning the patient next to the device and installing the patient’s hand; Overall: Combined duration. The dashed red line indicates the maximal duration of 5 min that therapists indicated in our survey [84] were willing to spend for the setup

The duration required by the therapists for the setup is depicted in Fig. 11. To change the handle, it took therapists 74.0 (62.5–102.5) s and for the actual patient setup 168.0 (111.5–192.5) s. This results in an overall setup duration of 233.0 (201.5–272.0) s. The range was large, with one therapist only requiring 86 s while another one needing 382 s.

All patients except two trained with both games until the end. One participant (P6) asked to stop the cocktail bar game after 07:27 min and the slingshot game after 04:43 min. Another participant (P7) stopped the cocktail bar game after 08:32 min, but played the full 10:00 min of the slingshot game.

### Usability and usefulness questionnaires

**Fig. 12:**
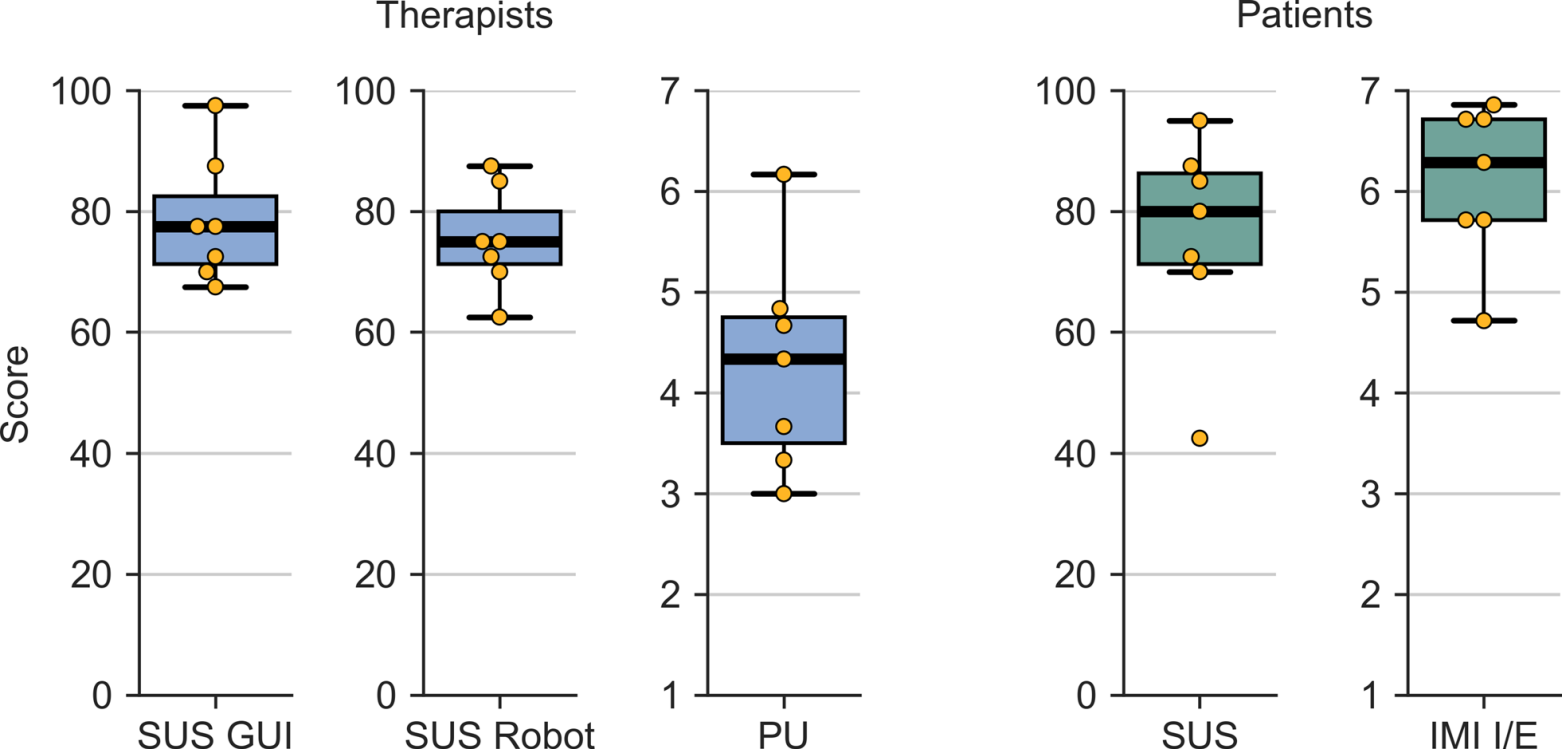
Results of the questionnaires. Left: Therapists’ scores are grouped in blue. SUS GUI: Therapists’ scores of SUS questionnaire on graphical user interface; SUS Robot: Therapists’ scores of SUS on robotic device; PU:, Therapists’ scores of the Perceived Usefulness questionnaire; Right: Patients’ scores are grouped in green. SUS: Patients’ scores of SUS questionnaire; IMI I/E: Patients’ scores of Interest/Enjoyment subscale of the IMI

The results of the questionnaires are visualized in Fig. 12. The therapists rated the usability of the GUI with a median SUS score of 77.5 (71.3–82.5) out of 100 and the usability of the robotic device with a SUS score of 75.0 (71.3–80.0) out of 100, indicating good to excellent usability for both according to [115]. The patients rated the usability of the therapy system with a mean SUS score of 80.0 (71.3–86.3) out of 100, also indicating good to excellent usability.

The score of the Perceived Usefulness subscale of the PUEU from the therapists was 4.3 (3.5–4.8) out of 1 to 7. Upon closer inspection, the relatively low score of this scale can be mostly explained by the results of the first question, “*Using the system in my job would enable me to accomplish tasks more quickly,*” and the second question, “*Using the system would improve my job performance.*” They were the lowest rated items on this scale with a score of 3 (2.5–3.5) out of 1 to 7 for both. Interestingly, question six of this questionnaire, “*I would find the system useful in my job*,” was rated with 6 (6–7). Per-question box plots of the questionnaire results can be found in Additional file 2.

The enjoyment of the therapy session with the robotic device was rated with a median score of the IMI Enjoyment/Interest subscale of 6.3 (5.7–6.7) out of 1 to 7. Patient P3, who rated the lowest usability score with 42.5 and the lowest Enjoyment/Interest score with 4.7, reported a general dislike of computers and computer games during the subsequent semi-structured interview.

### Semi-structured interviews

Here, we report the findings of the thematic analysis, listed according to the resulting themes. Exemplary statements are marked with (T) and (P) for therapists and patients, respectively. Some words were added in parentheses to the quotes to provide context and improve readability.

#### Overall experience

 The general feedback was very positive. The therapists and patients liked the device and the games. The therapists mentioned that the device is easy to use and that the games are motivating. The patients liked the games and the haptic feedback. They also mentioned that the device is simple to use and that they would like to use it again.

*“I had the feeling that it is quite mature. It was fun to practice.”*(P2)

*“I think it is very nice and motivates patients.”*(T4)

#### Ease of use

 The therapists highlighted that the setup of the device is not complicated and that the adjustments are made relatively quickly. However, they mentioned that better instructions for the positioning of the patient’s chair with respect to the robotic device are needed.

*“Quite simple to install. Should go quite quickly, even with spasticity.”*(T6)

*“The system is reasonably compact and reasonably quick to set up. The distance to the chair was critical.”*(T3)

*“It was a pleasant experience. I was installed relatively quickly.”*(P1)

It was also mentioned that the GUI was easy to use. In particular, therapists liked the logical flow and found that the right amount of settings and buttons was available. However, the settings for assistive forces and game difficulty were not always clear, especially how the plus and minus buttons for adjusting the assistive forces are mapped to the anatomic directions. The use of the real-time plot was only reported by three therapists, who appreciated it but would like more time to develop a better intuition in order to fully benefit from it. They liked that the plot allows them to better understand the involvement and effort of the patient. For example, without the plot, it can sometimes be difficult to tell if a patient is actually actively trying to grasp a liquid dispenser in the cocktail bar game, especially if the involved forces are low.

*“I liked the logic and structure. I could work through one thing at a time, like a checklist. Everything was there and clear. It didn’t go into too much unnecessary detail.”*(T3)

*“(After the experiment) I still have a mess with the plus and minus buttons.”*(T2)

*“The curves are interesting to see. Otherwise, it’s difficult (to see what’s happening) just by watching the patient’s hand. I might need a little more time to interpret the direction of the force plots.”*(T6)

#### Comfort and fingertip slipping

 This theme was predominantly present when we asked participants about possible improvements. Here, the majority of the participants reported that their fingertips slipped out of the fingertip fixation at some point. Further, minor adaptations of the thumb fixation (e.g., the strap should not go over the interphalangeal joint, add a second strap), the wrist support (prolong it slightly in the proximal direction), and the materials (softer handle, softer support) were requested.

*“The finger slipped out. In both games, this was a drawback.”*(T1)

*“I think the dorsal finger fixation is actually good, but the patient still slipped out.”*(T3)

*“Nothing bothered me, it was comfortable.”*(P7)

*“It is not compatible with my hands. Maybe you should have something for short-fingered people.”*(P4)

#### Clinical application

 In general, therapists reported in the semi-structured interviews that they considered the rehabilitation system useful for therapy. It was appreciated that the games are of stimulative nature (i.e., attracting patients’ focus and encouraging active participation) and that they kept the patients focused during the selected duration.

*“It would be very nice if this would find its way into therapy, assuming it is not too expensive. I think it will complement physiotherapy very well.”*(T4)

*“The games have the advantage of having something appealing for all generations. Cocktail bar for younger, slingshot for older people. It is good that the games are repetitive but still always change a little while playing.”*(T2)

*“I like the fact that different movement sequences are required. In the case of the cocktail bar, I liked that there are different variations of the task. The games have a very good challenging character.”*(T7)

Therapists also noticed that the system could work well in group therapy, potentially after making the robot more rugged. It was also mentioned that a dedicated GUI only for the patients could be useful.

*“Once everything is set, you could let patients train themselves. Could be used well in group therapy.”*(T4)

*“I would welcome such a device for group therapy. (For this,) a better enclosure for the robot is missing.”*(T5)

#### Transferability to ADL

 The potential transferability of the skills learned during training to ADL was, in general, estimated to be good. Two factors seem to contribute to this. First, the available movements and ranges of motion were mostly considered to be appropriate, especially the independent thumb movement was mentioned positively. Therapists mentioned that adding pronosupination and movements in the vertical direction would allow training in picking up objects.

*“There are all important thumb movements, which is something very special. There are moments in which the pronosupination position does not correspond to natural gripping. For example, I would grip a ball from above (with a pronated wrist). For that, you would need an additional degree of freedom for pronosupination.”*(T4)

*“I could imagine that the (skill) transfer can succeed. The task with the cocktail bar illustrates this well, as it gives the patient an idea of how to grab a bottle or something similar. The Slingshot game perhaps contributes a little less.”*(T1)

Second, the haptic rendering was very well perceived. In fact, all participants except one (P4: *“I felt nothing”*) complimented the physical interaction with the virtual game elements and liked the natural and realistic feeling. Therapists emphasized the importance of sensory information for motor learning. Here, the interaction with the cocktail bar dispensers was particularly appreciated.

*“This (the haptic rendering) is very important. The feedback you get is something very essential. Without this feedback, there is probably no motor rehabilitation.”*(T1)

*“The (sensory) input is important and makes it more real. It’s important from a therapeutic point of view because it’s more comparable to everyday life. It is good and important that it is really like when you want to grasp some real objects.”*(T2)

*“I liked this feedback very much. I also found it very positive that the hand could be pressed on an object when approaching. The feedback is almost realistic.”*(P2)

*“The force dosage is always an issue (in everyday life), which is also taken into account by the robot.”*(T7)

*“It was a natural interaction. There was a realistic feeling. I felt that I was grasping something.”*(P1)

#### Future developments

 Apart from improving the aforementioned drawbacks, it was mentioned that the mechanics and software could be made more robust. During experiments with two patients (P6 and P7), instabilities in the physics engine occurred and the system had to be restarted. The safety mechanisms worked well and prevented any abrupt movements or forces, but it was mentioned that this should be improved in the future. One therapist also mentioned that the difference between the haptic sensations in the cocktail bar game could be even more pronounced. Finally, the addition of auditory feedback was suggested.

*“It was a pity that it had to be restarted once. I have doubts if it is (mechanically) stable enough for frequent use.”*(T6)

*“The difference between the bottles in the cocktail bar game could be even stronger.”*(T3)

*“Auditory feedback would be desirable for the games.”*(T5)

### Handle sizes and hand dimensions

Overall, including all 14 participants, the handle size *S* was chosen six times, *SM* five times, *ML* two times, and *L* only once. Smaller handle sizes were therefore preferred, which could indicate that the handles were, in general, too large despite having been designed based on anthropomorphic databases.

The mean and standard deviation of the hand measurements are reported in Table 4, together with the pooled means derived from the measurements of Garrett [96, 97] and finger length conversion with the scaling factors of Buchholz [95] that we used in the design of the handles. The hand lengths of our study participants were significantly shorter by 9.0 mm and the finger lengths were significantly shorter by 20.6 mm compared to the pooled means. The hand breadth was not significantly different.

**Table 4:** Results from the paired *t*-test comparison of hand dimensions as measured in our study with the reference values used in the device development. The values in brackets indicate the respective standard deviation

Group	Hand length (mm)	Hand breadth (mm)	Finger length (mm)
Reference [95–97]	186.7 (12.52)	82.2 (7.38)	101.5 (6.81)
Our study	177.7 (11.86)	82.0 (7.30)	81.0 (10.95)
Difference	− 9.0 ($p=.009$)	− 0.2 ($p=0.92$)	− 20.6 ($p<.001$)

## Discussion

### From clinical requirements to clinical testing

We presented the user-centered development and clinical usability testing of a novel haptic sensorimotor upper-limb rehabilitation system that leverages comprehensive somatosensory information through whole-hand haptic rendering. Based on an initial assessment of clinical requirements, we co-created our solution together with clinical personnel to maximize system usability, resulting in an end-effector-style device for a simple setup. Our robotic device consists of a commercial delta robotic base (three translational DoFs) with a custom hand module (three DoFs), providing a unique combination of DoFs that allows the training of clinically relevant functional reach and grasp movements [84]. The only adjustment required between patients is swapping the handle of the hand module. Moreover, the hand module can retract to a compact cylindrical-shaped configuration during setup while still providing full-range finger flexion/extension and thumb circumduction motion, which, to our knowledge, has not been achieved in haptic rehabilitation devices to date. To provide meaningful ecologically valid haptic rendering during reach and grasp tasks, we devised a physics engine-based approach, where we can simulate arbitrary interactions between the patient’s hand and virtual tangible objects in engaging computer games.

To evaluate the clinical feasibility of our device, we conducted a usability study with 14 end-users, comprising both therapists and patients. After an introductory session, therapists exercised with one of their daily stroke patients using our robotic system. In a mixed-methods approach, we collected quantitative and qualitative data for a comprehensive usability assessment. We identified key elements to be maintained, enhanced, or newly incorporated in our device and to be considered for future developments in the field of robotic neurorehabilitation.

### Insights from the clinical usability study

#### The device design was perceived as well-conceived and useful

The device design was generally regarded as thoughtful and adequate for clinical upper-limb rehabilitation after stroke with minor suggestions for improvement, e.g., the addition of pronosupination movements, even though this would lead to a more complex device design. It must be noted that our device’s workspace (i.e., translational RoM) is smaller than the ones of the most comparable devices, the Gentle/G or the CyberTeam [71, 73]. However, a larger workspace would again have resulted in a considerably more complicated device as it would likely have required a mechanism to allow for roll, pitch and yaw movements of the hand module for larger movements. Our system currently provides six (or five with blocked *z*-axis) DoF to train realistic and complex tasks with great flexibility. This demands that patients coordinate multiple degrees of freedom during exercises, which can be challenging for patients in the initial stages of rehabilitation. Yet, finding the optimal number and choice of DoF is subject to ongoing research [116] and is likely also influenced by the impairment level of the patient [117].

Nevertheless, the therapists’ appreciation of the available DoF endorsed our design choices that followed from the assessment of clinical requirements. Our robotic device, and the hand module in particular, seems to provide a reasonable set of DoFs with sufficient RoM to allow functional grasping without abundant functionalities that would further complexify the device. The therapists’ perception that the selected DoF and RoM are appropriate also aligns with existing literature.

It has been suggested that functionally more relevant outcomes can be achieved by focusing on the rehabilitation of distal limbs [118, 119]. Given that the relative covered RoM of the hand is large compared to similar devices, while the workspace is comparably small, our device indeed slightly emphasizes distal movements. The combination of our device’s proximal and distal DoFs and their corresponding RoM is unique, warranting further investigation of its impact on therapy outcomes and skill transferability to real-life ADL.

Technology acceptance models [110, 120], suggest that the ease of use and perceived usefulness are important predictors of whether users will use a new technology when they are presented with it. When it comes to the perceived usefulness of our device by therapists, the results from different questions from the Perceived Usefulness (PU) questionnaire are mixed. It is remarkable that when directly asked if they would find the device useful in their job (question Q6, see PU questionnaire in Additional file 2), therapists’ answers were consistently very positive. However, the answers to the questions related to time savings or their performance in their job seem to be more neutral. We must acknowledge that “performance” was not further specified and was left to the participants’ interpretation. The fact that therapists do not foresee time savings compared to conventional therapy might be inherent to robotic devices that—like our device—require some sort of setup or adjustments. Yet, setting up the device took therapists, on average, less than five minutes, despite not having much practice, i.e., they only set up the examiner’s hand once before the therapy session. It is not clear if therapists also considered the potential overall time savings per patient and resulting potential increased efficiency when using our device in group therapy, as suggested by therapist T4. Moreover, note that the PU consisted of only positive statements. A low rating shows, therefore, disagreement with the positive statement, but it does not automatically imply agreement with a negative version of the statement. Nevertheless, these findings point out areas of improvement that need to be further investigated and addressed.

#### The system was regarded as easy to use

All three usability questionnaires (SUS) pointed to good to excellent usability from the perspectives of both patients and therapists, with median scores between 75.0 and 80.0 out of 100. As a comparison, the three-DoF HandyBot scored 76.3 overall and 85.0 for its GUI [58]. The two-DoF ReHapticKnob’s user interface scored 85.0, and its two games were rated at 76.3 and 68.8 [121]. The PLUTO with one actuated DoF and two passive DoF scored 85.0 [122]. The developers of the one-DoF ETH MIKE for finger proprioception therapy reported an overall score of 73.0. These figures illustrate our device’s competitive positioning in usability within haptic robotic neurorehabilitation devices.

The device’s good to excellent usability is further supported by the overall setup duration, which was below five minutes—our requirement for a quick setup [84]—except for one therapist who required 6 min and 22 s. We are confident that this time would be further reduced with slightly more practice. We can not compare this setup time to other haptic rehabilitation devices as this information is usually not reported in literature. Yet, we also learned from the therapists’ responses in the semi-structured interviews that the positioning of the patient with respect to the robot (or vice-versa) was not clear. We acknowledge that explicit instructions were missing and will include them in future practice.

Importantly, therapists commended our achievements in making training with the device accessible for patients with hypertonicity and spasticity through the compact handle during patient setup and the adjustable assistive forces. This observation is particularly relevant as muscle hypertonicity and spasticity are prevalent in approximately one third of stroke patients [123–125]

When it comes to the GUI, therapists’ statements revealed that the favorable usability score may partly stem from the provided logical flow, thus aligning with the Gestalt principles in user interface design [126]. The reportedly clear overall structure might have outweighed minor inconveniences like the ambiguous button labels in the settings tab. Although integrating sensor feedback in robotic therapy systems is recommended to overcome the gap between the therapist and patient’s body that robotic devices may create [127], the use of the force plot was only reported by three therapists. It seemed like therapists found it interesting but were not sure how to interpret it in the experimental patient session. Hence, a clearer design and/or enhancing user familiarity through additional practice or improved instructions could further increase the usefulness of the GUI and, thus, utilization of the robotic device’s full potential.

#### The robotic system and advanced haptic rendering enable functional training

High-quality haptic rendering conveys essential sensory information during object grasping and fine manipulation, potentially leading to improved motor control and learning [30, 44, 128, 129]. To quote one of the therapists in this study: “*(the haptic rendering) is very important. The feedback you get is something very essential. Without this feedback, there is probably no motor rehabilitation.*” The semi-structured interviews indeed revealed that the whole-hand haptic rendering was appreciated by both therapists and patients. It has been mentioned that the interactions between the hand and the objects feel intuitive and predictable. Nevertheless, it was pointed out that the difference in the haptic effects during the grasping of the different liquid dispensers could be more accentuated. It could indeed be useful to make the intensity or characteristics of the haptic effects—such as the stiffnesses and viscosities in the cocktail bar game—explicitly adjustable by the therapists to better suit the individual patient’s level of sensorimotor impairment.

Interestingly, one patient (P2) who was not able to perform a complete self-initiated opening of the hand discovered that it was possible to only partially open the hand and then press the fingers against a virtual object to assist the hand opening. It is important to highlight that this does not fall under haptic guidance as encountered in literature [130, 131]. Instead, our haptic rendering provided the user with close-to-realistic (although we do not claim complete realism) congruent visuo-haptic sensations to encourage naturalistic self-initiated hand-object interactions during reaching and grasping. The aforementioned example raises the question of whether and how training tasks with such whole-hand haptics could increase the transferability of the skills learned in robotic interventions to relevant skills for real-life ADL tasks.

Related to transferability and naturalistic interactions, the desire for auditory feedback was brought up in the interviews. Although auditory cues were already requested in our initial requirements assessment [84], we did not integrate this into our game because we wanted to avoid too many components at once in this first usability study. However, as it was requested again in the semi-structured interview, it should be considered in future developments. Indeed, following the multiple-resource theory [132], multi-modal sensory information can increase motor performance [77, 133]. However, such a feature should be optional as the provision of audiovisual multi-modal information in an already cognitively demanding situation—in stroke rehabilitation, depending on the individual patient’s abilities—can also undesirably excessively increase the cognitive workload [77].

#### The virtual training tasks are highly enjoyable

The high IMI Interest/Enjoyment score of 6.3 out of 7.0 and the positive feedback of the patients regarding the games indicate that the patients’ experience not only with the robot but also with both games was enjoyable. A similar conclusion was drawn by Colombo et al., who found an IMI Interest/Enjoyment result of 6.0 in their robot-assisted rehabilitation game [20]. The general enjoyment of the games is further supported by the fact that the majority of patients played the games for the full ten minutes. The short gameplay of patient P6 could likely be explained by the patient’s reported experience with virtual environments, robotics, and haptics due to his experience in building virtual reality racing simulators. The quick understanding of how the rehabilitation system works may have led to the decision to discontinue the games after a few minutes despite the positive feedback and high interest that P6 reported during the subsequent interview.

An exception to this was patient P3, who communicated a general dislike of computers and computer games. Although it is tempting to ignore such fundamentally technology-averse attitudes, they must be considered when designing technological solutions with equal access to all. Further research is needed to understand how robotic rehabilitation can be made accessible and enjoyable also for patients with low computer and e-health literacy. This also highlights the importance of personalization (and adaptation) in robotic neurorehabilitation. It has been shown that the human-robot interaction and the resulting motor performance in virtual sensorimotor tasks are modulated by personality traits [134]. While therapist T2 praised our games—“*The games have the advantage of having something appealing for all generations.... It is good that the games are repetitive, but still always change a little while playing*”— further personalization of the games and haptic effects might be required to unfold the full potential of our therapy system.

Finally, we also observed a tendency for increased focus of the patients as the therapy session progressed, potentially entering the state of flow—i.e., full involvement in the activity to the point of forgetting their surroundings [135]. Therapists corroborated this observation, yet further investigation with flow questionnaires will be required to validate this [136]. If such an increase in patients’ focus on the game rather than their own bodily movements could be confirmed, a positive impact on motor learning outcomes due to the increased external focus of attention and motivation can also be expected according to the OPTIMAL theory [137].

#### Designing for large ranges of motion for diverse hand shapes is a challenge

One of the main identified usability issues is that the patients’ fingers tended to slip out of the fingertip fixation. In our hand module, the fingers are not fixated on the device between the MCP joint and the distal segment, enabling our device’s distinct characteristics, such as the compact handle during setup and the large RoM, without the complicated adjustments of a hand exoskeleton design. This leads to an inherently increased risk of the fingers slipping out of their fixation compared to a design where each finger segment is fully constrained. However, even authors of comparable devices with smaller RoM also reported similar issues when working with stroke patients, highlighting the challenge of finding the right compromise between usability, RoM, adjustability, and ease of setup [121].

Related to this, we observed a preference for smaller handle sizes and that participants’ hand dimensions were generally shorter than anticipated during the design phase. There are multiple possible explanations for these observations: i) Although we used the same anatomical landmarks as Garrett [96, 97] and Buchholz [95], we might have introduced measurement errors. Especially the measurements of the finger length in our study could be prone to errors since Buchholz measured from the MCP joint center, which is not easy to locate in vivo. ii) The scaling values that we used to compute the finger lengths in the design phase were derived from only six cadavers by Buchholz and may thus lack the reliability that would have been needed for our application. iii) The hands of our participants were indeed shorter than the average reported by Garrett. Although i) cannot be excluded, it is likely that we overestimated the finger lengths during our development due to ii), leading to an overall increased risk of slipping out of the finger fixation, which was then further aggravated by iii). Consequently, participants naturally tended to choose smaller handles to partially counteract this issue.

When it comes to the thumb flexion/extension, we observed that it was only minimally used by the participants. We acknowledge that the chosen mechanism with the circular approximation of the thumb tip path during flexion/extension is indeed sub-optimal for larger thumb flexion/extension movements as the corresponding thumb tip path is insufficiently approximated [85] and thus restricted the RoM in software. However, humans also tend to first align their hand with respect to the thumb during reaching and then move the fingers with respect to the thumb [138, 139]. This—in combination with the absence of negative feedback on the thumb flexion/extension—indicates that the ultimately available RoM might still have been sufficient and adequate.

### Study limitations

Our study also has some limitations and shortcomings. First, in the scope of this study, it was not possible to assess the effect of our device or haptic rendering algorithm on sensorimotor (re)learning in stroke patients. Although our conclusions are based on professionals’ opinions, the transferability of our haptic exercises to ADL remains thus speculative. A longitudinal study will be required to make well-informed statements about the effects on skill learning and rehabilitation outcomes when training with our device and whole-hand haptic rendering.

Another limitation of our study is the lack of standardized assessment of the participant’s sensorimotor ability (e.g., Fugl-Meyer, Barthel-Index, or box and block test), which would have given us a first indication regarding the range of disabilities for which our system might be suited. Moreover, assessment of the participants’ hand spasticity would have allowed a more informed discussion of the compact cylindrical handle during setup. Unfortunately, such additional assessments were not possible due to constraints in the clinical routines.

We can assume that we identified the major usability issues with a total of 14 participants interacting with our system, as it has been shown that five participants allow detecting approximately 85% of usability problems, and studies with ten participants cover approximately 95% on average [140]. Nevertheless, the thoroughness of the usability evaluation could have been further improved by also recording and assessing the therapists’ behavior and interaction with the system objectively and in finer granularity. This could, for example, be achieved through eye-tracking or video recordings without substantially prolonging the experimental sessions [61, 141, 142].

Finally, another limitation of our development is that we could not perform formal intermediate testing with patients during the development, as we were legally bound and did not have ethical approval for the prototypes. For future developments, we highly recommend an approach where intermediate prototypes are to be admitted by the responsible ethical committee for testing with patients. Yet, significant design modifications between iterations may necessitate amendments or re-submission of the ethical application, introducing time-consuming administrative processes. If the field of robotic neurorehabilitation aims to bring deeply user-driven designs into clinical practice, addressing this challenge in future developments is essential. On the same note, in retrospect, we would embrace a more standardized method in performing the co-creation sessions, like the think-aloud method [143] or the cognitive walk-through method [144].

### Future work

Our next step is to address imminent usability issues of our system such as the fingertips slipping out of their fixation or the ambiguous adjustments of the assistive forces. Moreover, the integration of our device with stereoscopic visualization (e.g., with a head-mounted display [106]) could enable the full utilization of the z-axis for 3D movements. In terms of software, we will also further improve the robustness of our physics engine-based haptic rendering approach, e.g., through formally derived stability criteria.

In future work, we also aim to investigate the efficiency and long-term effectiveness of whole-hand sensorimotor training. Thereby, the investigation of the long-term transferability of skills learned in whole-hand interactions in virtual environments to ADL with healthy and, more critically, stroke patients is essential. The long-term transfer of sensorimotor skills acquired through robotic and virtual training tasks to real-life tasks is generally not well investigated yet [145, 146]. However, our newly developed system promises to be a pivotal tool for such investigations as it has the potential to alleviate the persisting gap between the complex dynamics of real-life objects and simplified dynamics of virtual objects during reach and grasp tasks [147].

A related aspect to be further investigated is the design of virtual therapy games. In robotic rehabilitation, games often mimic situations of ADL, such as cooking, cleaning, or gardening [26, 148], even though these activities might not actually be motivating for all patients [149]. This motivates the development of games that are not directly related to ADL, but still mimic movement patterns and somatosensory information during common ADL involving reaching and grasping. While we completely abstained from visually replicating ADL, other studies suggested using a mix of ADL-related tasks with enjoyable and fun games [128, 150]. It remains, therefore, to be investigated how mirroring only the sensomotoric experience of ADL—particularly with systems like ours that provide naturalistic haptic interactions—affects motivation and therapy outcomes compared to exercises that also visually resemble ADL. Ultimately, this might also be governed by the patient’s personal preferences and personality traits [134].

Alongside the development of personalized games and haptic effects, the development of automatic assistance (e.g., guidance-as-needed [151]) or difficulty adjustment algorithms presents another direction for future research. For this study, we only implemented a manually adjustable constant-force assistance for each DoF to not confound the usability of our system by the potential interference in the perception between the haptic rendering and the adaptive assistance. In future studies, the device could be equipped with skin stretch actuators [152, 153] that target different mechanoreceptors to mitigate this and better differentiate assistance from haptic rendering. While various sophisticated assist-as-needed frameworks have been suggested recently [154, 155], the optimal assistance framework for our system still needs to be defined as the interaction of high-fidelity haptic rendering and assistive forces should be considered [30]. Based on our experience from this study, it will be particularly important to not only automatically adjust the assistance and game difficulty in an optimal manner but also to account for therapists’ online inputs in such algorithms.

### Conclusion

Based on clinical requirements, we developed and tested a novel upper-limb rehabilitation system that allows for naturalistic hand-object interactions in virtual training tasks. Our development compromises a robotic device, algorithms for haptic feedback, two games designed for haptic rehabilitation, and a graphical therapist interface. Our device is one of the first to enable the training of reach and grasp movements with meaningful haptic information on fingers, hand and arm.

To test the feasibility of therapy with our system, we performed a clinical usability study with seven therapists and seven stroke patients. We found a high appreciation of the haptic information that was conveyed through the training from both therapists and patients. The main issue that we identified was that the fingertips of some patients tended to slip out of the device. When we compared patients’ and therapists’ hand dimensions with our assumptions based on anthropomorphic databases during the development, we found that the participants’ hands were smaller than anticipated, contributing to this issue. Nevertheless, the games, the robotic device, and the graphical user interface were generally perceived as well-thought.

We believe that this kind of device can provide patients access to clinical robotic interventions with meaningful, diverse, and naturalistic haptic sensations. Such devices have the potential to increase the transferability of skills learned in virtual exercises to ADL. This might finally enable robotic rehabilitation to unfold its full potential, improve the efficacy of neurorehabilitation in the face of the upcoming challenges from expected staff shortages and rising stroke prevalence, and improve the lives of stroke patients.

## Supplementary Information

Supplementary file 1.

Supplementary file 2.

## Data Availability

Not applicable.

## References

[1] Feigin VL, et al. World Stroke Organization (WSO): global stroke fact sheet 2022. Int J Stroke. 2022;17:18–29. 34986727 10.1177/17474930211065917

[2] Guidetti S, Ytterberg C, Ekstam L, Johansson U, Eriksson G. Changes in the impact of stroke between 3 and 12 months post-stroke, assessed with the stroke impact scale. J Rehabilit Med. 2014;46:963–8. 10.2340/16501977-1865 25188837

[3] Kwakkel G, Kollen BJ, Van der Grond JV, Prevo AJ. Probability of regaining dexterity in the flaccid upper limb: Impact of severity of paresis and time since onset in acute stroke. Stroke. 2003;34:2181–6. 12907818 10.1161/01.STR.0000087172.16305.CD

[4] Parker VM, Wade DT, Hewer RL. Loss of arm function after stroke: Measurement, frequency, and recovery. Disabil Rehabilit. 1986;8:69–73. 10.3109/03790798609166178 3804600

[5] Lai S-M, Studenski S, Duncan PW, Perera S. Persisting consequences of stroke measured by the stroke impact scale. Stroke. 2002;33:1840–4. 10.1161/01.STR.0000019289.15440.F2. 12105363 10.1161/01.str.0000019289.15440.f2

[6] Wade DT, Langton-Hewer R, Wood VA, Skilbeck CE, Ismail HM. The hemiplegic arm after stroke: measurement and recovery. J Neurol Neurosurg Psychiat. 1983;46:521–4. 10.1136/jnnp.46.6.521. 6875585 10.1136/jnnp.46.6.521 PMC1027442

[7] Mayo NE, Wood-Dauphinee S, Côté R, Durcan L, Carlton J. Activity, participation, and quality of life 6 months poststroke. Archiv Phys Med Rehabilit. 2002;83:1035–42. 10.1053/apmr.2002.33984 12161823

[8] Lotze M. Motor learning elicited by voluntary drive. Brain. 2003;126:866–72. 10.1093/brain/awg079. 12615644 10.1093/brain/awg079

[9] Tollár J, et al. High frequency and intensity rehabilitation in 641 subacute ischemic stroke patients. Archiv Phys Med Rehabilit. 2021;102:9–18. 10.1016/j.apmr.2020.07.012 32861668

[10] French B, et al. Repetitive task training for improving functional ability after stroke. Cochrane Database Syst Rev. 2016. 10.1002/14651858.CD006073.pub3. 27841442 10.1002/14651858.CD006073.pub3 PMC6464929

[11] Kleim JA, Barbay S, Nudo RJ. Functional reorganization of the rat motor cortex following motor skill learning. J Neurophysiol. 1998;80:3321–5. 9862925 10.1152/jn.1998.80.6.3321

[12] Grefkes C, Grefkes C, Fink GR, Fink GR. Recovery from stroke: current concepts and future perspectives. Neurol Res Pract. 2020;2:17. 33324923 10.1186/s42466-020-00060-6 PMC7650109

[13] Foerch C, Misselwitz B, Sitzer M, Steinmetz H, Neumann-Haefelin T. The projected burden of stroke in the German federal state of Hesse up to the year 2050. Deutsches Ärzteblatt int. 2008;105:467–73. 10.3238/arztebl.2008.0467. 10.3238/arztebl.2008.0467 PMC2696913 19626195

[14] World Health Organization. Global strategy on human resources for health: workforce 2030. Geneva: World Health Organization; 2016.

[15] Lo AC, et al. Robot-assisted therapy for long-term upper-limb impairment after stroke. New Engl J Med. 2010;362:1772–83. 10.1056/NEJMoa0911341. 20400552 10.1056/NEJMoa0911341 PMC5592692

[16] Gassert R, Dietz V. Rehabilitation robots for the treatment of sensorimotor deficits: a neurophysiological perspective. J NeuroEng Rehabilit. 2018;15:46. 10.1186/s12984-018-0383-x. 10.1186/s12984-018-0383-x PMC5987585 29866106

[17] Zhang C, Li-Tsang CW, Au RK. Robotic approaches for the rehabilitation of upper limb recovery after stroke. Int J Rehabilit Res. 2017;40:19–28. 10.1097/MRR.0000000000000204 27926617

[18] Alarcón-Aldana AC, Callejas-Cuervo M, Bo APL. Upper limb physical rehabilitation using serious videogames and motion capture systems: a systematic review. Sensors. 2020;20:1–22. 10.3390/s20215989 PMC7660052 33105845

[19] Chen M-H, et al. A controlled pilot trial of two commercial video games for rehabilitation of arm function after stroke. Clin Rehabilit. 2015;29:674–82. 10.1177/0269215514554115. (**PMID: 25322868**). 10.1177/0269215514554115 25322868

[20] Colombo R, et al. Design strategies to improve patient motivation during robot-aided rehabilitation. J NeuroEng Rehabilit. 2007;4:3. 10.1186/1743-0003-4-3. 10.1186/1743-0003-4-3 PMC1805445 17309790

[21] Calabrò RS, et al. Does hand robotic rehabilitation improve motor function by rebalancing interhemispheric connectivity after chronic stroke? Encouraging data from a randomised-clinical-trial. Clin Neurophysiol. 2019;130:767–80. 30904771 10.1016/j.clinph.2019.02.013

[22] Lee BO, Saragih ID, Batubara SO. Robotic arm use for upper limb rehabilitation after stroke: a systematic review and meta-analysis. Kaohsiung J Med Sci. 2023;39:435–45. 36999894 10.1002/kjm2.12679 PMC11895899

[23] Ranzani R, et al. Neurocognitive robot-assisted rehabilitation of hand function: a randomized control trial on motor recovery in subacute stroke. J NeuroEng Rehabilit. 2020;17:1–13. 10.1186/s12984-020-00746-7 PMC7444058 32831097

[24] Mehrholz J, Pohl M, Platz T, Kugler J, Elsner B. Electromechanical and robot-assisted arm training for improving activities of daily living, arm function, and arm muscle strength after stroke. Cochrane Database Syst Rev. 2015. 10.1002/14651858.CD006876.pub4. 26559225 10.1002/14651858.CD006876.pub4 PMC6465047

[25] Rodgers H, et al. Robot assisted training for the upper limb after stroke (RATULS): a multicentre randomised controlled trial. Lancet. 2019;394:51–62. 10.1016/S0140-6736(19)31055-4. 31128926 10.1016/S0140-6736(19)31055-4 PMC6620612

[26] Klamroth-Marganska V, et al. Three-dimensional, task-specific robot therapy of the arm after stroke: a multicentre, parallel-group randomised trial. Lancet Neurol. 2014;13:159–66. 10.5167/uzh-88799. 24382580 10.1016/S1474-4422(13)70305-3

[27] Bolognini N, Russo C, Edwards DJ. The sensory side of post-stroke motor rehabilitation. Restorat Neurol Neurosci. 2016;34:571–86. 10.3233/RNN-150606 PMC5605470 27080070

[28] Handelzalts S, et al. Integrating tactile feedback technologies into home-based telerehabilitation: opportunities and challenges in light of COVID-19 pandemic. Front Neurorobot. 2021;15: 617636. 33679364 10.3389/fnbot.2021.617636 PMC7925397

[29] Piggott L, Wagner S, Ziat M. Haptic neurorehabilitation and virtual reality for upper limb paralysis: a review. Crit Rev Biomed Eng. 2016;44:1–32. 27652449 10.1615/CritRevBiomedEng.2016016046

[30] Özen Ö, Buetler KA, Marchal-Crespo L. Towards functional robotic training: motor learning of dynamic tasks is enhanced by haptic rendering but hampered by arm weight support. J NeuroEng Rehabilit. 2022;19:19. 10.1186/s12984-022-00993-w. 10.1186/s12984-022-00993-w PMC8842890 35152897

[31] Pettypiece CE, Goodale MA, Culham JC. Integration of haptic and visual size cues in perception and action revealed through cross-modal conflict. Exp Brain Res. 2010;201:863–73. 19949777 10.1007/s00221-009-2101-1

[32] Scott SH. Optimal feedback control and the neural basis of volitional motor control. Nat Rev Neurosci. 2004;5:532–44. 15208695 10.1038/nrn1427

[33] Binkofski F, Kunesch E, Classen J, Seitz RJ, Freund H-J. Tactile apraxia: Unimodal Apractic disorder of tactile object exploration associated with parietal lobe lesions. Brain. 2001;124:132–44. 10.1093/brain/124.1.132. 11133793 10.1093/brain/124.1.132

[34] Grundmann A, Hardwick M, Ledingham D, Miller J. Sensory ataxia with cranial nerve palsies. Pract Neurol. 2022;22:85–9. 10.1136/practneurol-2021-003044. 34135093 10.1136/practneurol-2021-003044

[35] Carey LM. Somatosensory loss after stroke. Crit Rev Phys Rehabilit Med. 1995;7:51–91.

[36] Bernard-Espina J, Beraneck M, Maier MA, Tagliabue M. Multisensory integration in stroke patients: a theoretical approach to reinterpret upper-limb proprioceptive deficits and visual compensation. Front Neurosci. 2021;15: 646698. 33897359 10.3389/fnins.2021.646698 PMC8058201

[37] Welmer AK, Holmqvist LW, Sommerfeld DK. Limited fine hand use after stroke and its association with other disabilities. J Rehabilit Med. 2008;40:603–8. 10.2340/16501977-0218 19020692

[38] Meyer S, Karttunen AH, Thijs V, Feys H, Verheyden G. How do somatosensory deficits in the arm and hand relate to upper limb impairment, activity, and participation problems after stroke? Syst Rev Phys Ther. 2014;94:1220–31. 10.2522/ptj.20130271. 10.2522/ptj.20130271 24764072

[39] Carey LM, Matyas TA, Baum C. Effects of Somatosensory Impairment on Participation After Stroke. The American Journal of Occupational Therapy. 2018;72:72032051001–720320510010. 10.1177/0269215508090674. 10.5014/ajot.2018.025114 PMC5915232 29689179

[40] Zandvliet SB, Kwakkel G, Nijland RH, van Wegen EE, Meskers CG. Is recovery of somatosensory impairment conditional for upper-limb motor recovery early after stroke? Neurorehabil Neural Repair. 2020;34:403–16. 32391744 10.1177/1545968320907075 PMC7222963

[41] Salisbury K, Brock D, Massie T, Swarup N, Zilles C. Haptic rendering: Programming touch interaction with virtual objects. *Proc Symp Interact 3D Graph* 1995;123–130.

[42] Metzger J-C, Lambercy O, Gassert R. High-fidelity rendering of virtual objects with the ReHapticKnob - novel avenues in robot-assisted rehabilitation of hand function. *2012 IEEE Haptics Symposium (HAPTICS)* 2012;51–56. http://ieeexplore.ieee.org/document/6183769/.

[43] Galofaro E, et al. Bimanual motor strategies and handedness role in human-robot haptic interaction. IEEE Trans Haptics. 2023;16:296–310. 37167042 10.1109/TOH.2023.3272698

[44] Özen Ö, Buetler KA, Marchal-Crespo L. Promoting motor variability during robotic assistance enhances motor learning of dynamic tasks. Front Neurosci. 2021. 10.3389/fnins.2020.600059/full. 10.3389/fnins.2020.600059 PMC7884323 33603642

[45] Zimmermann Y, Sommerhalder M, Wolf P, Riener R, Hutter M. ANYexo 2.0: a fully actuated upper-limb exoskeleton for manipulation and joint-oriented training in all stages of rehabilitation. IEEE Trans Robot. 2023;39:2131–50.

[46] Sommerhalder M, Zimmermann Y, Cizmeci B, Riener R, Hutter M. Physical human-robot interaction with real active surfaces using haptic rendering on point clouds. IEEE International Conference on Intelligent Robots and Systems. 2020;9767–73.

[47] Krebs HI, Hogan N, Aisen ML, Volpe BT. Robot-aided neurorehabilitation. Tech Rep. 1998;6:75. 10.1109/86.662623 PMC2692541 9535526

[48] Bionik Laboratories Corp. *InMotion*^®^*ARM/HAND*. 2024. https://bioniklabs.com/inmotion-arm-hand/. Accessed 24 Jan 2024.

[49] Howard IS, Ingram JN, Wolpert DM. A modular planar robotic manipulandum with end-point torque control. J Neurosci Methods. 2009;181:199–211. 19450621 10.1016/j.jneumeth.2009.05.005

[50] Qian C, et al. Quantitative assessment of motor function by an end-effector upper limb rehabilitation robot based on admittance control. Appl Sci. 2021;11:6854.

[51] Articares. *H-Man*. 2024. https://articares.com/h-man/. Accessed 24 Jan 2024.

[52] Fourier Intelligence. *ArmMotus™M2 Pro*. 2024. https://fourierintelligence.com/armmotus-m2-pro/. Accessed 24 Jan 2024.

[53] Barrett Medical. *Burt*^®^. 2024. https://medical.barrett.com/. Accessed 24 Jan 2024.

[54] Fourier Intelligence. *ArmMotus™EMU*. 2024. https://fourierintelligence.com/emu/. Accessed 24 Jan 2024.

[55] Rätz R, Conti F, Müri RM, Marchal-Crespo L. A novel clinical-driven design for robotic hand rehabilitation: combining sensory training, effortless setup, and large range of motion in a palmar device. Front Neurorobot. 2021;15:1–22. 10.3389/fnbot.2021.748196/full. 10.3389/fnbot.2021.748196 PMC8721892 34987371

[56] Chen PN, Chen YT, Hsiu H, Chen RJ. The application of an impedance-passivity controller in haptic stability analysis. Appl Sci. 2021;11:1–13.

[57] Metzger J-C, Lambercy O, Chapuis D, Gassert R. Design and characterization of the ReHapticKnob, a robot for assessment and therapy of hand function. *2011 IEEE/RSJ International Conference on Intelligent Robots and Systems* 2011;3074–3080. http://ieeexplore.ieee.org/document/6094882/.

[58] Ranzani R, et al. Design, characterization and preliminary usability testing of a portable robot for unsupervised therapy of hand function. Front Mech Eng. 2023;8:1–17.

[59] Taheri H, et al. Design and preliminary evaluation of the FINGER rehabilitation robot: controlling challenge and quantifying finger individuation during musical computer game play. J NeuroEng Rehabil. 2014;11:10. 10.1186/1743-0003-11-10. 24495432 10.1186/1743-0003-11-10 PMC3928667

[60] Van Damme, N, Ratz R, Marchal-Crespo L. Towards unsupervised rehabilitation: development of a portable compliant device for sensorimotor hand rehabilitation. *IEEE International Conference on Rehabilitation Robotics* **2022-July**, 2022;25–29. 10.1109/ICORR55369.2022.9896556 36176098

[61] Rätz R, Ratschat AL, Cividanes-Garcia N, Ribbers GM, Marchal-Crespo L. Designing for usability: development and evaluation of a portable minimally-actuated haptic hand and forearm trainer for unsupervised stroke rehabilitation. Front Neurorobot. 2024;18:1351700. 10.3389/fnbot.2024.1351700 10.3389/fnbot.2024.1351700 PMC11024237 38638360

[62] Pezent E, Rose CG, Deshpande AD, O’Malley MK. Design and characterization of the OpenWrist: a robotic wrist exoskeleton for coordinated hand-wrist rehabilitation. *2017 International Conference on Rehabilitation Robotics (ICORR)* 2017;720–725. https://ieeexplore.ieee.org/document/8009333/. 10.1109/ICORR.2017.8009333 28813905

[63] Sarac M, Solazzi M, Sotgiu E, Bergamasco M, Frisoli A. Design and kinematic optimization of a novel underactuated robotic hand exoskeleton. Meccanica. 2016;52:749–61. 10.1007/s11012-016-0530-z.

[64] Agarwal P, Deshpande AD. Subject-specific assist-as-needed controllers for a hand exoskeleton for rehabilitation. IEEE Robot Automat Lett. 2018;3:508–15.

[65] Lambelet C, et al. Characterization and wearability evaluation of a fully portable wrist exoskeleton for unsupervised training after stroke. J NeuroEng Rehabil. 2020;17:1–17. 33028354 10.1186/s12984-020-00749-4 PMC7541267

[66] Van De Kamp C, Zaal FT. Prehension is really reaching and grasping. Exp Brain Res. 2007;182:27–34. 17516058 10.1007/s00221-007-0968-2

[67] Li Z, Gray K, Roldan JR, Milutinović D, Rosen J. The joint coordination in reach-to-grasp movements. IEEE International Conference on Intelligent Robots and Systems. 2014;906–11.

[68] Schwarz A, Veerbeek JM, Held JP, Buurke JH, Luft AR. Measures of interjoint coordination post-stroke across different upper limb movement tasks. Front Bioeng Biotechnol. 2021;8:1–17. 10.3389/fbioe.2020.620805 PMC7876346 33585418

[69] Pirondini E, et al. Evaluation of the effects of the Arm Light Exoskeleton on movement execution and muscle activities: a pilot study on healthy subjects. J NeuroEng Rehabil. 2016;13:1–21. 10.1186/s12984-016-0117-x. 26801620 10.1186/s12984-016-0117-x PMC4724067

[70] Buongiorno D, et al. WRES: A Novel 3 DoF WRist ExoSkeleton with tendon-driven differential transmission for neuro-rehabilitation and teleoperation. IEEE Robotics and Automation Letters. 2018;3:2152–9.http://ieeexplore.ieee.org/document/8304775/.

[71] Loureiro RC, Harwin WS. Reach & Grasp Therapy: design and control of a 9-DOF robotic neuro-rehabilitation system. *2007 IEEE 10th International Conference on Rehabilitation Robotics* 2007;00: 757–763. http://ieeexplore.ieee.org/document/4428510/.

[72] Zhu TL, Klein J, Dual SA, Leong TC, Burdet E, reachMAN2: a compact rehabilitation robot to train reaching and manipulation. *2014 IEEE/RSJ International Conference on Intelligent Robots and Systems* 2014;2107–2113. http://ieeexplore.ieee.org/document/6942845/.

[73] Ramirez-Zamora JD, *et al.* Kinematics modeling and experimental validation of a cyberforce haptic device based on passive control system. *2015 International Conference on Mechatronics, Electronics and Automotive Engineering (ICMEAE)* 2015;105–110. http://ieeexplore.ieee.org/document/7386203/.

[74] Loureiro RC, Harwin WS, Lamperd R, Collin C. Evaluation of reach and grasp robot-assisted therapy suggests similar functional recovery patterns on proximal and distal arm segments in sub-acute Hemiplegia. IEEE Trans Neural Syst Rehabili Eng. 2014;22:593–602. 10.1109/TNSRE.2013.2265263 23744701

[75] Colgate JE, Grafing PE, Stanley MC, Schenkel G. Implementation of stiff virtual walls in force-reflecting interfaces. *1993 IEEE Annual Virtual Reality International Symposium* 1993;202–208.

[76] Odermatt IA, et al. Congruency of information rather than body ownership enhances motor performance in highly embodied virtual reality. Front Neurosci. 2021;15: 678909. 34295219 10.3389/fnins.2021.678909 PMC8291288

[77] Sigrist R, Rauter G, Riener R, Wolf P. Augmented visual, auditory, haptic, and multimodal feedback in motor learning: a review. Psychonomic Bull Rev. 2013;20:21–53. 10.3758/s13423-012-0333-8 23132605

[78] Desmarais G, Meade M, Wells T, Nadeau M. Visuo-haptic integration in object identification using novel objects. Attent Percept Psychophys. 2017;79:2478–98. 10.3758/s13414-017-1382-x 28744702

[79] Tzafestas CS. Whole-hand kinesthetic feedback and haptic perception in Dextrous virtual manipulation. IEEE Trans Syst Man Cybernet Part A Syst Humans. 2003;33:100–13.

[80] Levac DE, Huber ME, Sternad D. Learning and transfer of complex motor skills in virtual reality: a perspective review. J NeuroEng Rehabil. 2019;16:121. 10.1186/s12984-019-0587-8. 31627755 10.1186/s12984-019-0587-8 PMC6798491

[81] Holt R, *et al.* User involvement in developing rehabilitation robotic devices: an essential requirement. *2007 IEEE 10th International Conference on Rehabilitation Robotics, ICORR’07* 2007;00: 196–204.

[82] Lu EC, et al. Development of a robotic device for upper limb stroke rehabilitation: a user-centered design approach. Paladyn J Behav Robot. 2012;2:176–84.

[83] Zanatta F, Giardini A, Pierobon A, Addario MD, Steca P. A systematic review on the usability of robotic and virtual reality devices in neuromotor rehabilitation : patients ’ and healthcare professionals ’ perspective. BMC Health Services Res. 2022. 10.1186/s12913-022-07821-w. 10.1186/s12913-022-07821-w PMC9020115 35443710

[84] Rätz R, Müri RM, Marchal-Crespo L. *Assessment of clinical requirements for a novel robotic device for upper-limb sensorimotor rehabilitation after stroke*. (eds Torricelli, D., Akay, M. & Pons, J. L.) *Converging Clinical and Engineering Research on Neurorehabilitation IV*, 171–175 (Springer International Publishing, Cham, 2022).

[85] Rätz R, Müri RM, Marchal-Crespo L. Design of a haptic palmar device with thumb flexion and circumduction movements for sensorimotor stroke rehabilitation. *44th Annual International Conference of the IEEE Engineering in Medicine & Biology Society (EMBC)* 2022;2644–2647. 10.1109/EMBC48229.2022.9871303 36085966

[86] Rätz R, Marchal-Crespo L. Physics engine-based whole-hand haptic rendering for sensorimotor neurorehabilitation. *IEEE World Haptics Conference (WHC)* 2023;279–285.

[87] Meyer JT, Gassert R, Lambercy O. An analysis of usability evaluation practices and contexts of use in wearable robotics. J NeuroEng Rehabil. 2021;18:1–16. 10.1186/s12984-021-00963-8. 34886902 10.1186/s12984-021-00963-8 PMC8656061

[88] McDonald S. Studying actions in context: a qualitative shadowing method for organizational research. Qualit Res. 2005;5:455–73. 10.1177/1468794105056923.

[89] Lee M, Rittenhouse M, Abdullah HA. Design issues for therapeutic robot systems: results from a survey of physiotherapists. J Intell Robot Syst Theory Appl. 2005;42:239–52.

[90] Lu EC, et al. The development of an upper limb stroke rehabilitation robot: identification of clinical practices and design requirements through a survey of therapists. Disabil Rehabil Assist Technol. 2011;6:420–31. 10.3109/17483107.2010.544370. 21184626 10.3109/17483107.2010.544370

[91] Hochstenbach-Waelen A, Seelen HA. Embracing change: practical and theoretical considerations for successful implementation of technology assisting upper limb training in stroke. J NeuroEng Rehabil. 2012;9:52. 10.1186/1743-0003-9-52. 22856548 10.1186/1743-0003-9-52 PMC3480833

[92] Nasr N, et al. The experience of living with stroke and using technology: opportunities to engage and co-design with end users. Disabil Rehabil Assist Technol. 2016;11:653–60. 10.3109/17483107.2015.1036469. 25879304 10.3109/17483107.2015.1036469

[93] Bullock IM, Zheng JZ, Rosa SDL, Guertler C, Dollar AM. Grasp frequency and usage in daily household and machine shop tasks. IEEE Trans Haptics. 2013;6:296–308. 24808326 10.1109/TOH.2013.6

[94] Feix T, Romero J, Schmiedmayer HB, Dollar AM, Kragic D. The GRASP taxonomy of human grasp types. IEEE Trans Human-Mach Syst. 2016;46:66–77.

[95] Buchholz B. Anthropometric data for describing the kinematics of the human hand. Ergonomics. 1992;35:261–73. 1572336 10.1080/00140139208967812

[96] Garrett JW. Anthropometry of the Air Force Female Hand. Air Force Aerospace Medical Research Lab, Wright-Patterson AFB, OH, USA: Tech. Rep; 1970.

[97] Garrett JW. Anthropometry of the hands of male Air Force flight personnel. Air Force Aerospace Medical Research Lab, Wright-Patterson AFB, OH, USA: Tech. Rep; 1970.

[98] Vergara M, Agost MJ, Gracia-Ibáñez V. Dorsal and palmar aspect dimensions of hand anthropometry for designing hand tools and protections. Human Factors Ergonom Manufact Service Indust. 2018;28:17–28. 10.1002/hfm.20714.

[99] Kapandji IA. *Physiology of the Joints, Volume 1, Upper Limb* 5th edition edn. 1982.

[100] Giurintano DJ, Hollister AM, Buford WL, Thompson DE, Myers LM. A virtual five-link model of the thumb. Med Eng Phys. 1995;17:297–303. 7633758 10.1016/1350-4533(95)90855-6

[101] Chang LY, Pollard NS. Method for determining kinematic parameters of the in vivo thumb carpometacarpal joint. IEEE Trans Biomed Eng. 2008;55:1897–906. 18595809 10.1109/TBME.2008.919854

[102] Bullock IM, Borras J, Dollar AM. Assessing assumptions in kinematic hand models: a review. *Proceedings of the IEEE RAS and EMBS International Conference on Biomedical Robotics and Biomechatronics* 2012;139–146.

[103] Coert JH, van Dijke GAH, Hovius SER, Snijders CJ, Meek MF. Quantifying thumb rotation during circumduction utilizing a video technique. J Orthopaedic Res. 2003;21:1151–5. 10.1016/S0736-0266(03)00114-1 14554232

[104] Lawrence D. Stability and transparency in bilateral teleoperation. IEEE Trans Robotics Automat. 1993;9:624–37.

[105] Garzás-Villar A. Game development for sensorimotor upper-limb rehabilitation after stroke. *Master’s thesis, University of Bern, Switzerland*. 2022.

[106] Wenk N, Buetler KA, Penalver-Andres J, Müri RM, Marchal-Crespo L. Naturalistic visualization of reaching movements using head-mounted displays improves movement quality compared to conventional computer screens and proves high usability. J NeuroEng Rehabil. 2022;19:1–24. 10.1186/s12984-022-01101-8. 36494668 10.1186/s12984-022-01101-8 PMC9733395

[107] Just F, et al. Human arm weight compensation in rehabilitation robotics: efficacy of three distinct methods. J NeuroEng Rehabil. 2020;17:1–18. 32024528 10.1186/s12984-020-0644-3 PMC7003349

[108] Flash T, Hogan N. The coordination of arm movements: an experimentally confirmed mathematical model. J Neurosci. 1985;5:1688–703. 10.1523/JNEUROSCI.05-07-01688.1985. 4020415 10.1523/JNEUROSCI.05-07-01688.1985 PMC6565116

[109] Brooke J. SUS: A ’Quick and Dirty’ Usability Scale. *Usability Evaluation In Industry* 1996;207–212. https://www.taylorfrancis.com/books/9781498710411/chapters/10.1201/9781498710411-35.

[110] Davis FD. Perceived usefulness, perceived ease of use, and user acceptance of information technology. MIS Quart Manag Inform Syst. 1989;13:319–39.

[111] Sivan M, et al. Home-based computer assisted arm rehabilitation (HCAAR) robotic device for upper limb exercise after stroke: results of a feasibility study in home setting. J NeuroEng Rehabil. 2014;11:1. 25495889 10.1186/1743-0003-11-163 PMC4280043

[112] McAuley ED, Duncan T, Tammen VV. Psychometric properties of the intrinsic motivation inventoiy in a competitive sport setting: a confirmatory factor analysis. Res Quart Exercise Sport. 1989;60:48–58. 10.1080/02701367.1989.10607413 2489825

[113] Braun V, Clarke V. Qualitative research in psychology using thematic analysis in psychology using thematic analysis in psychology. Qualitat Res Psychol. 2006;3:77–101.

[114] Bernhardt J, et al. Agreed definitions and a shared vision for new standards in stroke recovery research: the stroke recovery and rehabilitation roundtable taskforce. Int J Stroke. 2017;12:444–50. 10.1177/1747493017711816. 28697708 10.1177/1747493017711816

[115] Bangor A, Kortum PT, Miller JT. An empirical evaluation of the system usability scale. Int J Human-Comput Interact. 2008;24:574–94.

[116] Penalver-Andres J, *et al.* Do we need complex rehabilitation robots for training complex tasks? *IEEE International Conference on Rehabilitation Robotics* **2019-June**, 2019;1085–1090. 10.1109/ICORR.2019.8779384 31374774

[117] Krebs HI, Saitoh E, Hogan N. Robotic therapy and the paradox of the diminishing number of degrees of freedom. Phys Med Rehabil Clin North Am. 2015;26:691–702. 10.1016/j.pmr.2015.06.003 PMC4630788 26522906

[118] Qian Q, et al. Distal versus proximal - an investigation on different supportive strategies by robots for upper limb rehabilitation after stroke: A randomized controlled trial. J NeuroEng Rehabil. 2019;16:1–16. 31159822 10.1186/s12984-019-0537-5 PMC6545723

[119] Hsieh YW, et al. Comparison of proximal versus distal upper-limb robotic rehabilitation on motor performance after stroke: A cluster controlled trial. Sci Reports. 2018;8:1–11. 10.1038/s41598-018-20330-3. 10.1038/s41598-018-20330-3 PMC5794971 29391492

[120] Morris Venkatesh, Davis Davis. User acceptance of information technology: toward a unified view. MIS Quarterly. 2003;27:425. 10.2307/30036540.

[121] Ranzani R, et al. Towards a platform for robot-assisted minimally-supervised therapy of hand function: design and pilot usability evaluation. Front Bioeng Biotechnol. 2021. 10.1101/2021.01.12.21249685. 33937218 10.3389/fbioe.2021.652380 PMC8082072

[122] Nehrujee A, *et al.* A plug-and-train robotic kit (PLUTO) for hand rehabilitation: pilot usability study. *Proceedings of the IEEE RAS and EMBS International Conference on Biomedical Robotics and Biomechatronics* **2020-Novem**, 2020;100–105.

[123] Urban PP, et al. Occurence and clinical predictors of spasticity after ischemic stroke. Stroke. 2010;41:2016–20. 10.1161/STROKEAHA.110.581991. 20705930 10.1161/STROKEAHA.110.581991

[124] Zeng H, Chen J, Guo Y, Tan S. Prevalence and risk factors for spasticity after stroke: a systematic review and meta-analysis. Front Neurol. 2021;11: 616097. 33551975 10.3389/fneur.2020.616097 PMC7855612

[125] Sommerfeld DK, Gripenstedt U, Welmer AK. Spasticity after stroke: an overview of prevalence, test instruments, and treatments. Am J Phys Med Rehabil. 2012;91:814–20. 22760104 10.1097/PHM.0b013e31825f13a3

[126] Liang Y. Application of Gestalt psychology in product human-machine Interface design. IOP Conf Series Mater Sci Eng. 2018;392: 062054. 10.1088/1757-899X/392/6/062054.

[127] Luciani B, Braghin F, Pedrocchi ALG, Gandolla M. Technology acceptance model for exoskeletons for rehabilitation of the upper limbs from therapists’ perspectives. Sensors. 2023;23:1–16. 10.3390/s23031721 PMC9919869 36772758

[128] Huang X, Naghdy F, Naghdy G, Du H. Clinical effectiveness of combined virtual reality and robot assisted fine hand motion rehabilitation in subacute stroke patients. IEEE International Conference on Rehabilitation Robotics. 2017;511–5. 10.1109/ICORR.2017.8009299 28813871

[129] Danion F, Diamond JS, Flanagan JR. The role of haptic feedback when manipulating nonrigid objects. J Neurophysiol. 2012;107:433–41. 10.1152/jn.00738.2011. 22013237 10.1152/jn.00738.2011

[130] Marchal-Crespo L, van Raai M, Rauter G, Wolf P, Riener R. The effect of haptic guidance and visual feedback on learning a complex tennis task. Exp Brain Res. 2013;231:277–91. 10.1007/s00221-013-3690-2. 24013789 10.1007/s00221-013-3690-2

[131] Liu J, Cramer SC, Reinkensmeyer DJ. Learning to perform a new movement with robotic assistance: Comparison of haptic guidance and visual demonstration. J NeuroEng Rehabil. 2006;3:1–10. 16945148 10.1186/1743-0003-3-20 PMC1569852

[132] Wickens CD. Multiple resources and performance prediction. Theoret Issues Ergonom Sci. 2002;3:159–77. 10.1080/14639220210123806.

[133] Pan L, Zhao L, Song A, Yin Z, She S. A novel robot-aided upper limb rehabilitation training system based on multimodal feedback. Front Robot AI. 2019;6:1–12. 33501117 10.3389/frobt.2019.00102 PMC7805779

[134] Garzás-Villar A, Boersma C, Derumigny A, Zgonnikov A, Marchal-Crespo L. The effect of haptic guidance during robotic-assisted motor training is modulated by personality traits. *2024 10th IEEE RAS/EMBS International Conference for Biomedical Robotics and Biomechatronics (BioRob)* 2024.

[135] van der Linden D, Tops M, Bakker AB. Go with the flow: a neuroscientific view on being fully engaged. Eur J Neurosci. 2021;53:947–63. 10.1111/ejn.15014. 33084102 10.1111/ejn.15014 PMC7983950

[136] Ottiger B, et al. Getting into a “Flow’’ state: a systematic review of flow experience in neurological diseases. J NeuroEng Rehabil. 2021;18:1–21. 10.1186/s12984-021-00864-w. 33879182 10.1186/s12984-021-00864-w PMC8059246

[137] Wulf G, Lewthwaite R. Optimizing performance through intrinsic motivation and attention for learning: the OPTIMAL theory of motor learning. Psychonom Bull Rev. 2016;23:1382–414. 10.3758/s13423-015-0999-9 26833314

[138] Wing AM, Fraser C. The contribution of the thumb to reaching movements. Quart J Exp Psychol Sect A. 1983;35:297–309. 10.1080/14640748308402135. 10.1080/14640748308402135 6571312

[139] Haggard P, Wing A. On the hand transport component of prehensile movements. J Motor Behav. 1997;29:282–7. 10.1080/00222899709600842 12453786

[140] Faulkner L. Beyond the five-user assumption: benefits of increased sample sizes in usability testing. Behav Res Methods Instrum Comput. 2003;35:379–83. 10.3758/BF03195514. 14587545 10.3758/bf03195514

[141] Goldberg JH, Wichansky AM. *Eye Tracking in Usability Evaluation* (Elsevier, 2003). https://linkinghub.elsevier.com/retrieve/pii/B978044451020450027X.

[142] Dittli J, et al. Mixed methods usability evaluation of an assistive wearable robotic hand orthosis for people with spinal cord injury. J NeuroEng Rehabil. 2023;20:162. 10.1186/s12984-023-01284-8. 38041135 10.1186/s12984-023-01284-8 PMC10693050

[143] Nielsen J, Clemmensen T, Yssing C. Getting access to what goes on in people’s heads? Proc Second Nordic conf Human-comput Interact. 2002;31:101–10. 10.1145/572020.572033.

[144] Beer T, Anodenko T, Sears A. A pair of techniques for effective interface evaluation: cognitive walkthroughs and think-aloud evaluations. Proc Human Factors Ergonomics Soc Ann Meet. 1997;41:380–4. 10.1177/107118139704100184.

[145] Basalp E, Wolf P, Marchal-Crespo L. Haptic training: Which types facilitate (re)learning of which motor task and for whom Answers by a review. *IEEE Transactions on Haptics* 2021;**1412**. 10.1109/TOH.2021.3104518 34388095

[146] Levac DE, Huber ME, Sternad D. Learning and transfer of complex motor skills in virtual reality: a perspective review. J NeuroEng Rehabil. 2019;16:1–15. 31627755 10.1186/s12984-019-0587-8 PMC6798491

[147] Zhang Z, Sternad D. Back to reality: differences in learning strategy in a simplified virtual and a real throwing task. J Neurophysiol. 2021;125:43–62. 33146063 10.1152/jn.00197.2020 PMC8087380

[148] Gorsic M, Tran MH, Novak D. Cooperative Cooking: A Novel Virtual Environment for Upper Limb Rehabilitation. *2018 40th Annual International Conference of the IEEE Engineering in Medicine and Biology Society (EMBC)* **2018-July**, 2018;3602–3605. https://ieeexplore.ieee.org/document/8513005/. 10.1109/EMBC.2018.8513005 PMC6320235 30441156

[149] Shah N, Amirabdollahian F, Basteris A. Designing motivational games for stroke rehabilitation. *2014 7th International Conference on Human System Interactions (HSI)* 2014;166–171. http://ieeexplore.ieee.org/document/6860468/.

[150] Frisoli A, et al. A randomized clinical control study on the efficacy of three-dimensional upper limb robotic exoskeleton training in chronic stroke. J NeuroEng Rehabil. 2022;19:1–15. 10.1186/s12984-022-00991-y. 35120546 10.1186/s12984-022-00991-y PMC8817500

[151] Marchal-Crespo L, McHughen S, Cramer SC, Reinkensmeyer DJ. The effect of haptic guidance, aging, and initial skill level on motor learning of a steering task. Exp Brain Res. 2010;201:209–20. 10.1007/s00221-009-2026-8. 19820920 10.1007/s00221-009-2026-8 PMC2832903

[152] Ratschat AL, Martín-Rodríguez R, Vardar Y, Ribbers GM, Marchal-Crespo L. Design and evaluation of a multi-finger skin-stretch tactile interface for hand rehabilitation robots. *2024 10th IEEE RAS/EMBS International Conference for Biomedical Robotics and Biomechatronics (BioRob)* 2024.

[153] Martín-Rodríguez R, Ratschat AL, Marchal-Crespo L, Vardar Y. Tactile weight rendering: a review for researchers and developers. In: IEEE Transactions on Haptics. 10.1109/TOH.2024.3453894 10.1109/TOH.2024.3453894 39226192

[154] Cho KH, Song WK. Robot-assisted reach training with an active assistant protocol for long-term upper extremity impairment poststroke: a randomized controlled trial. Archiv Phys Med Rehabil. 2019;100:213–9. 10.1016/j.apmr.2018.10.002. 10.1016/j.apmr.2018.10.002 30686326

[155] Pareek S, Nisar HJ, Kesavadas T. AR3n: A Reinforcement Learning-Based Assist-as-Needed Controller for Robotic Rehabilitation. *IEEE Robotics and Automation Magazine* 2023;1–8.

