# Supplementary material for: Enhancing stroke rehabilitation with whole-hand haptic rendering: development and clinical usability evaluation of a novel upper-limb rehabilitation device
Source: J Neuroeng Rehabil. 2024 Sep 27;21:172. doi: 10.1186/s12984-024-01439-1 (PMC11437669; doi:10.1186/s12984-024-01439-1)
Supplement: Supplementary file 1 [file 12984_2024_1439_MOESM1_ESM.pdf]

LAMBDA.3+

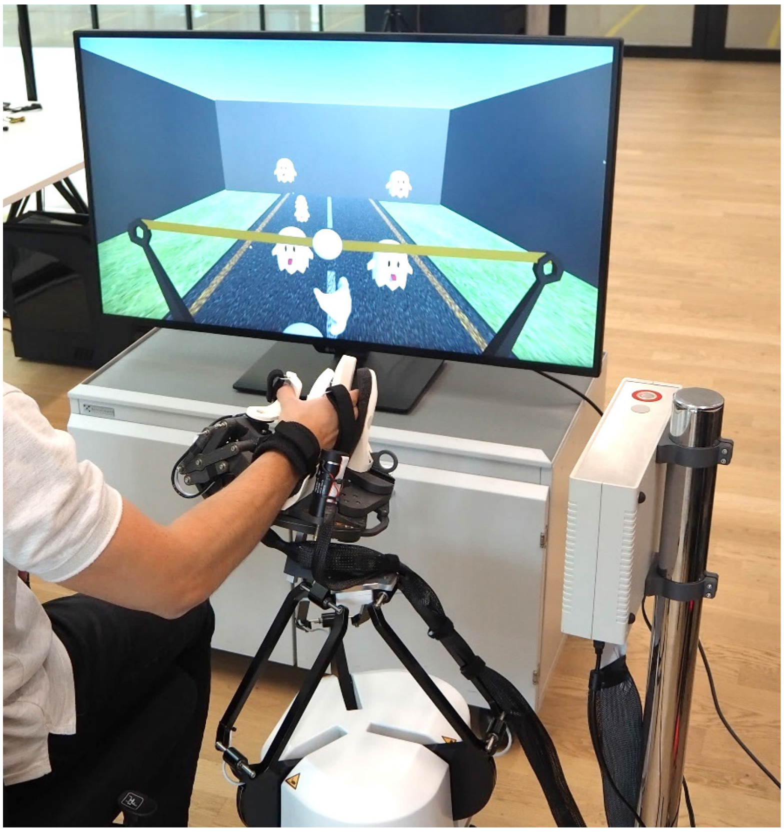

Version 1.0

QuickStart – Anleitung für Lambda.3+

Verantwortlich: Raphael Rätz, Karin Buetler, Laura Marchal-Crespo

Nur für klinische Studien

Motor Learning & Neurorehabilitation Laboratory
Kontakt: raphael.raetz@unibe.ch
Version: 14.03.2023 18:57

Übersicht

1) Roboter

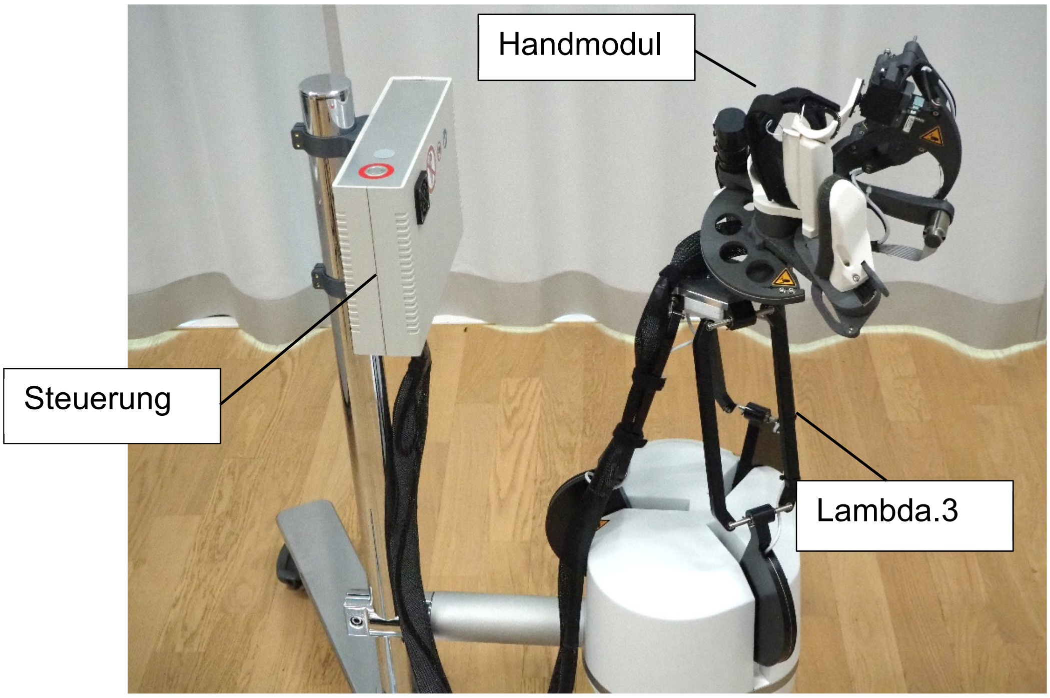

2) Handmodul

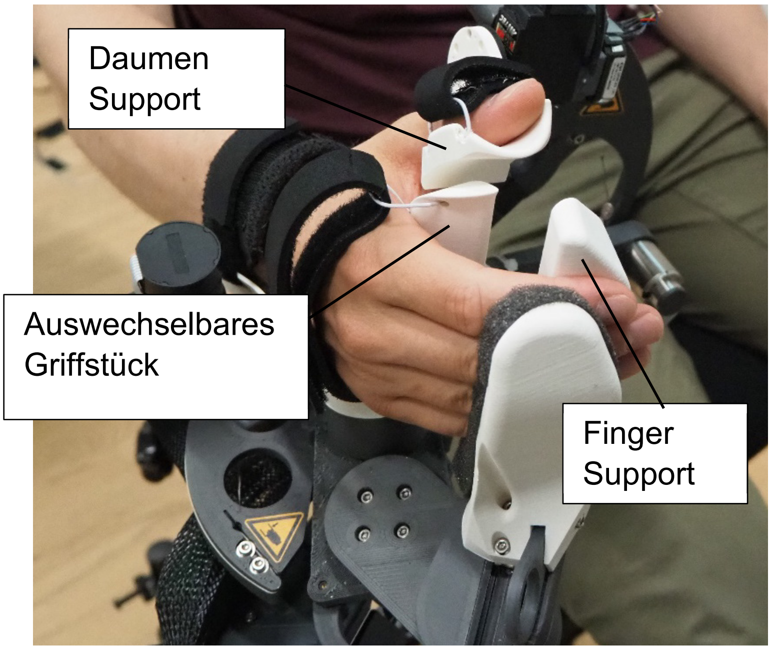

Motor Learning & Neurorehabilitation Laboratory
Kontakt: raphael.raetz@unibe.ch
Version: 14.03.2023 18:57

Einrichten

1) Räder Arretieren

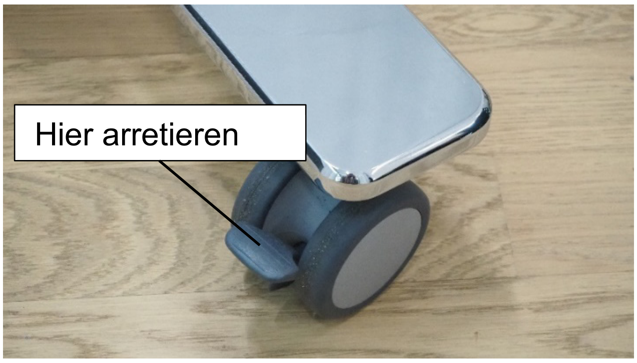

2) Roboter Starten

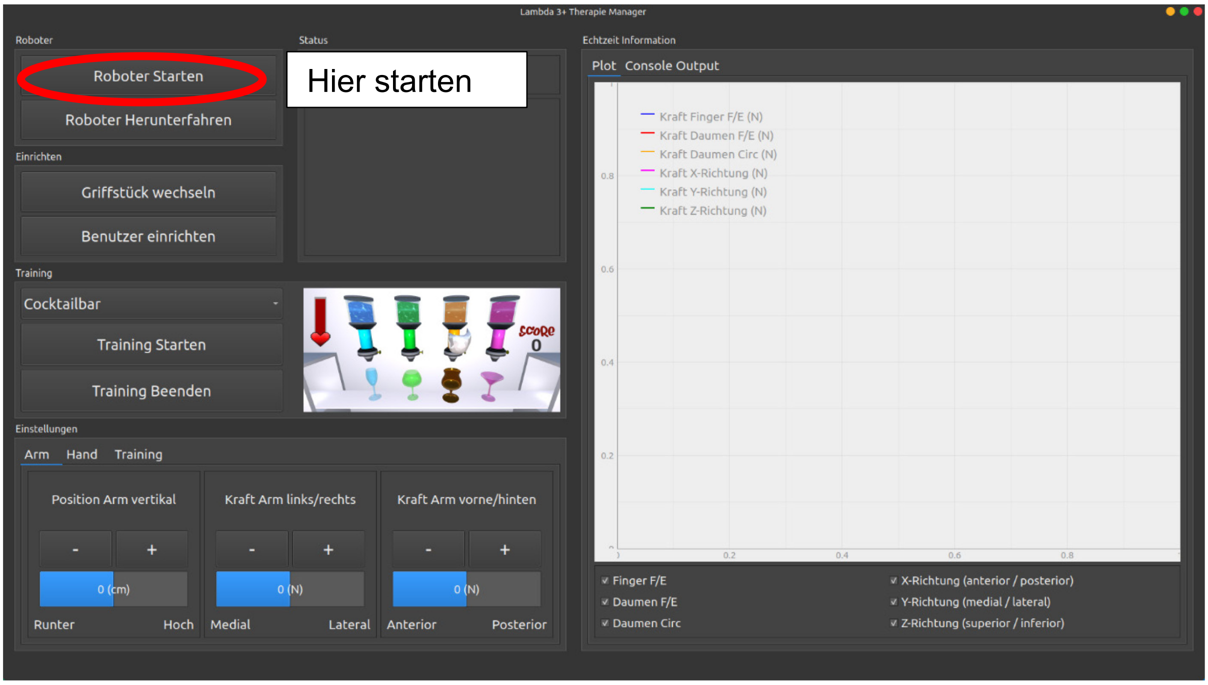

Motor Learning & Neurorehabilitation Laboratory
Kontakt: raphael.raetz@unibe.ch
Version: 14.03.2023 18:57

3) Griffstück auswechseln

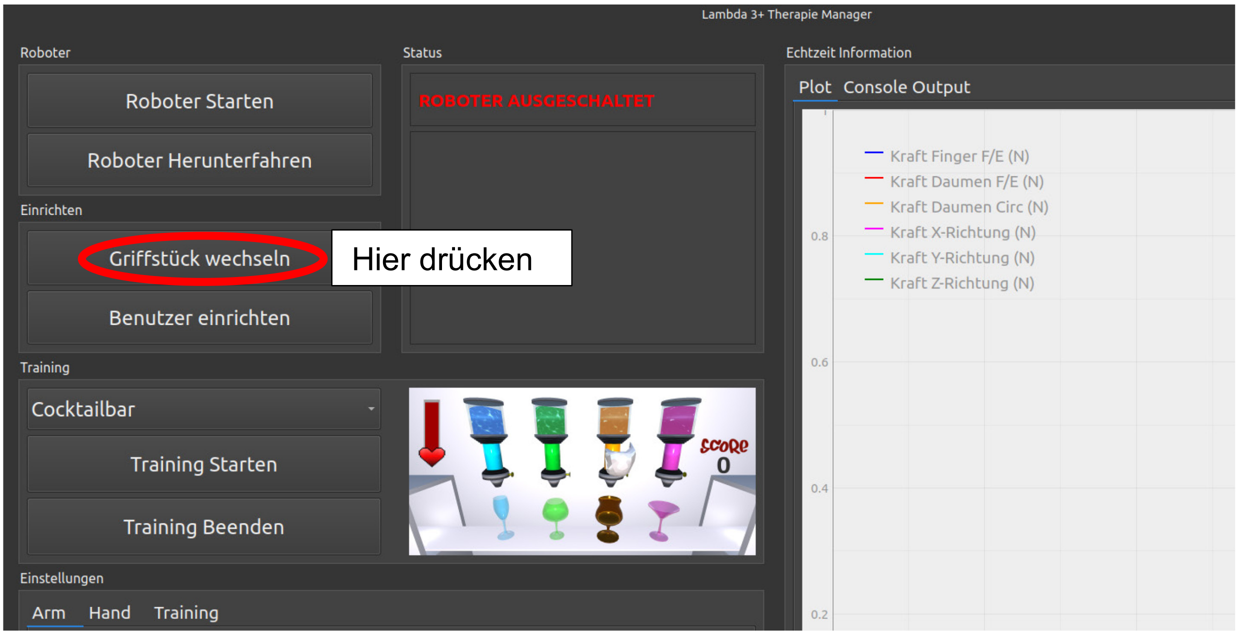

Handlänge (mm)	165 ± 10	180 ± 10	196 ± 10	211 ± 10
Empfohlene Grösse	S	SM	ML	L

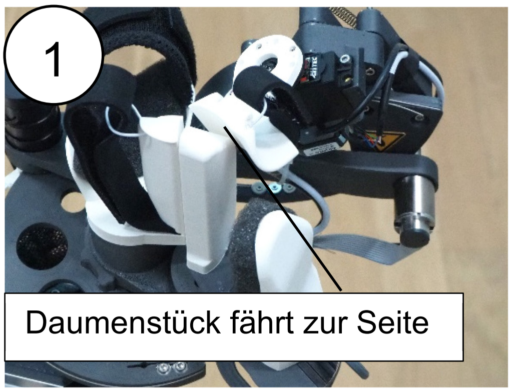

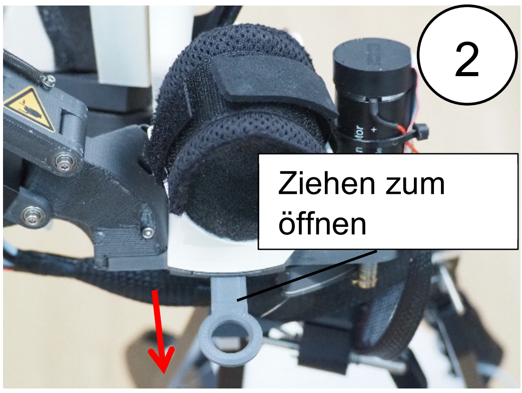

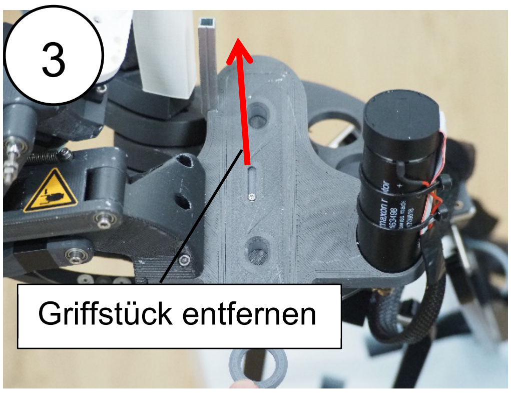

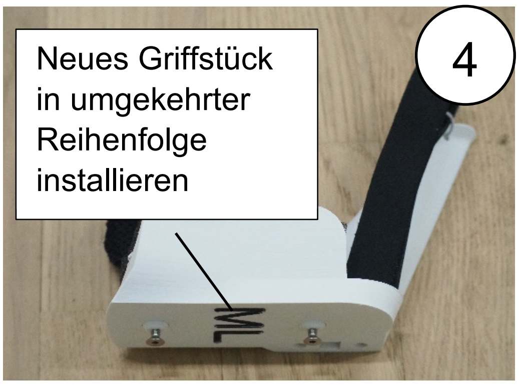

Motor Learning & Neurorehabilitation Laboratory
Kontakt: raphael.raetz@unibe.ch
Version: 14.03.2023 18:57

Benutzer Einrichten

1) Hand fixieren

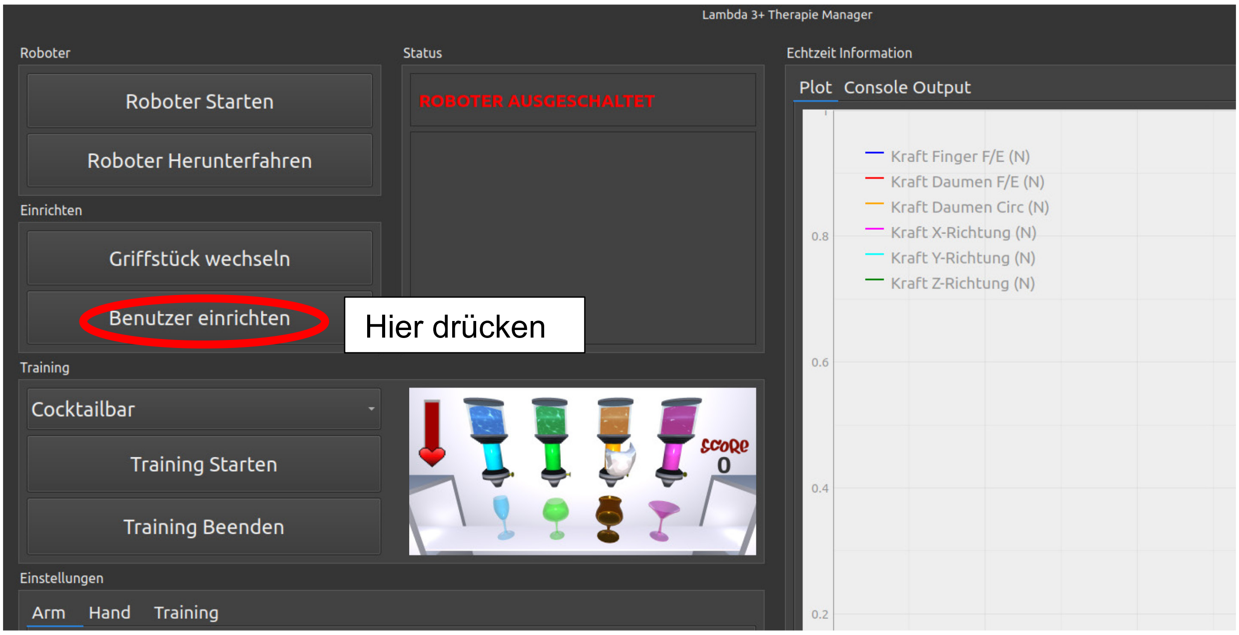

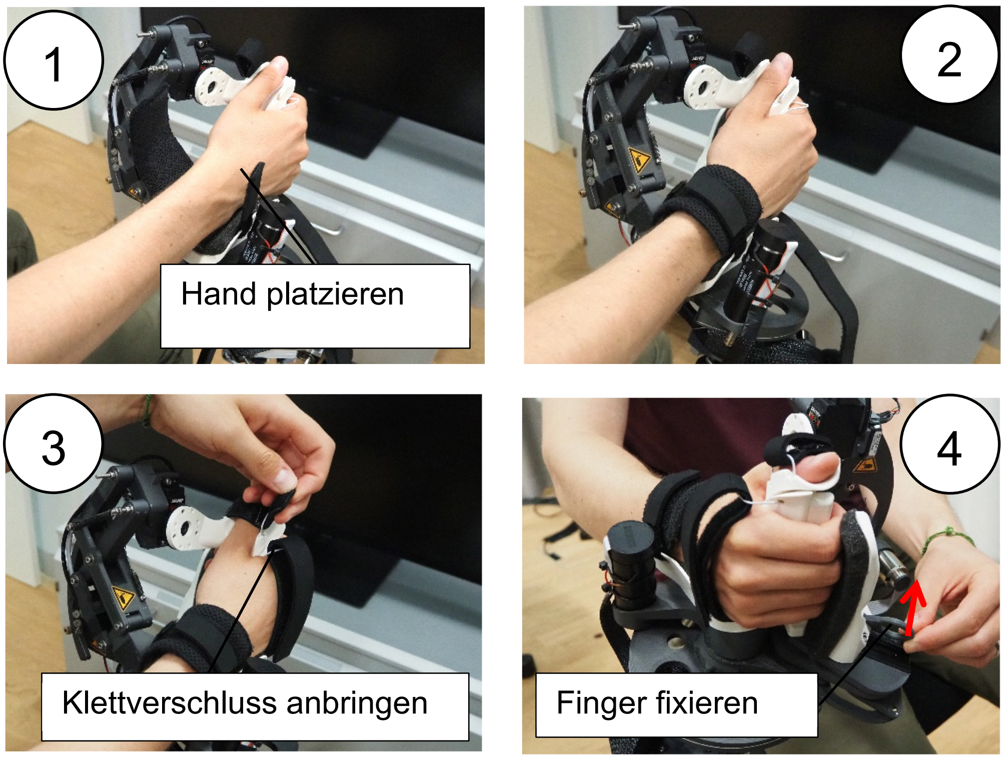

Motor Learning & Neurorehabilitation Laboratory
Kontakt: raphael.raetz@unibe.ch
Version: 14.03.2023 18:57

Betrieb & Notaus

1) Notaus

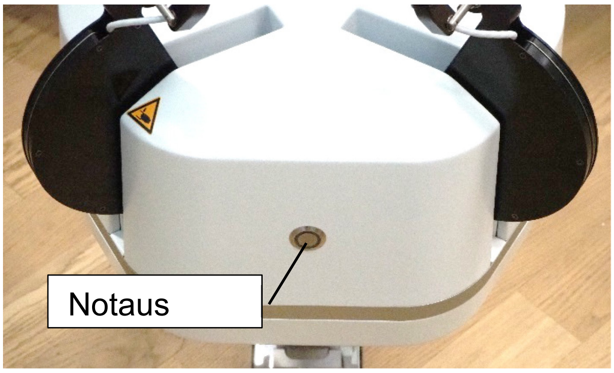

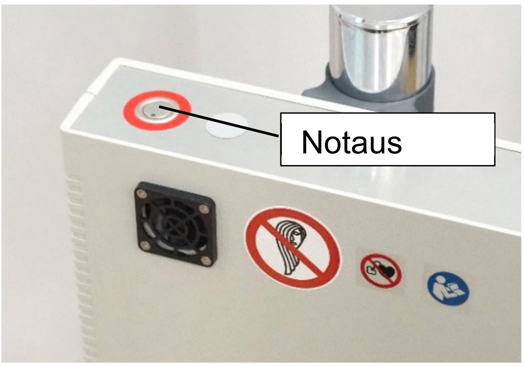

2) Spiel starten

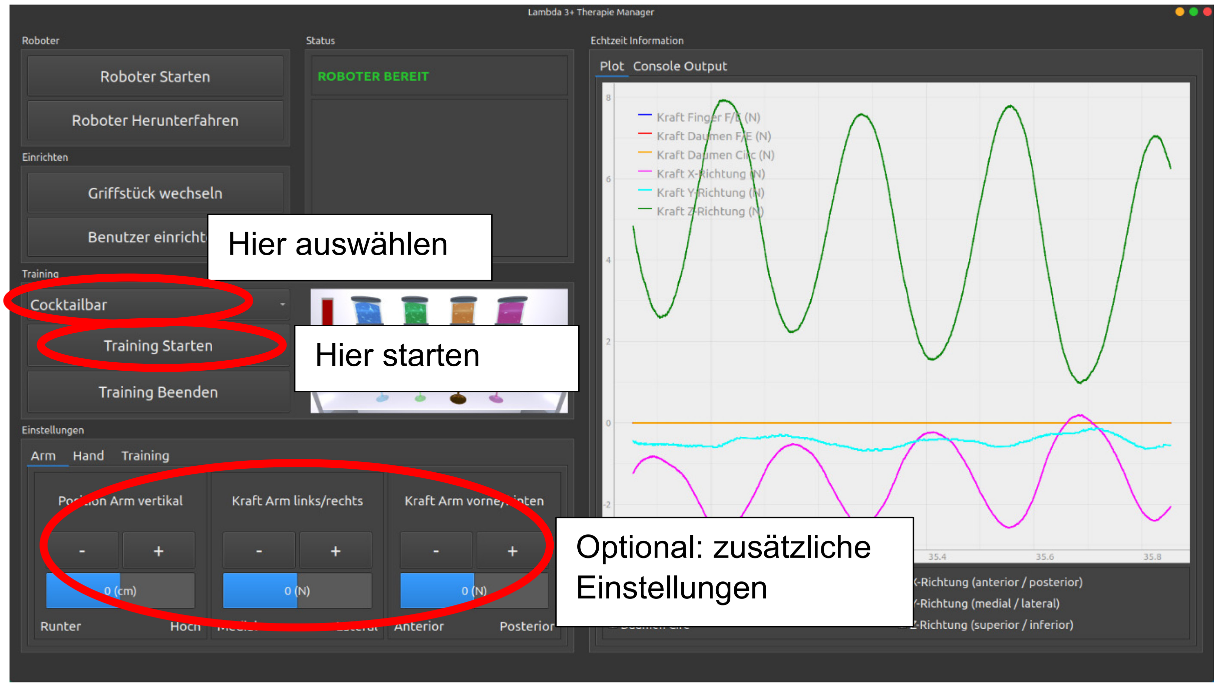

Motor Learning & Neurorehabilitation Laboratory
Kontakt: raphael.raetz@unibe.ch
Version: 14.03.2023 18:57

Sicherheitshinweise

1) Exklusionskriterien

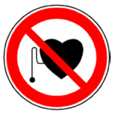

Patienten und Benutzer mit Herzschrittmachern, Insulinpumpen oder anderen medizinisch aktiven implantierbaren oder nicht-implantierbaren Geräten dürfen das System NICHT verwenden (wegen möglicher elektromagnetischer Störungen des Lambda.3+ Systems)

2) Zu Beachtende Stellen am Roboter

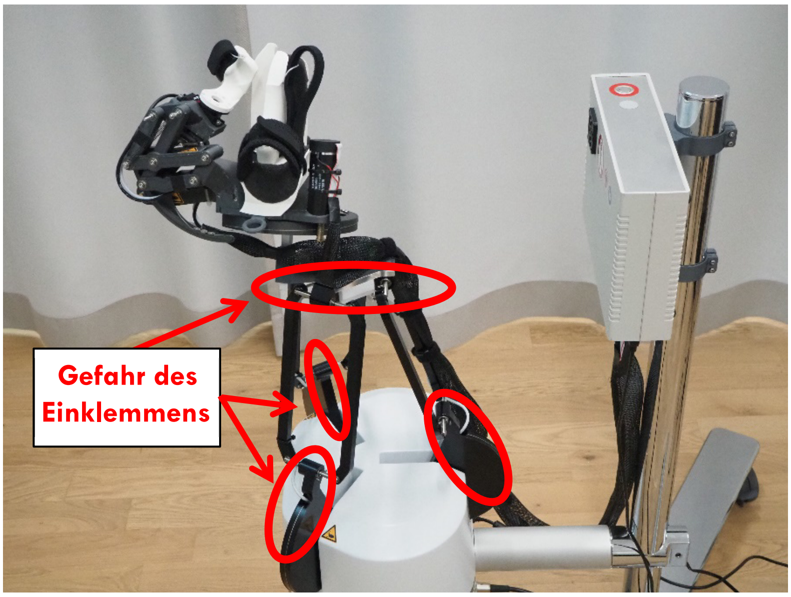

Figure 1 - Potenziell gefährliche Zonen am Lambda.3

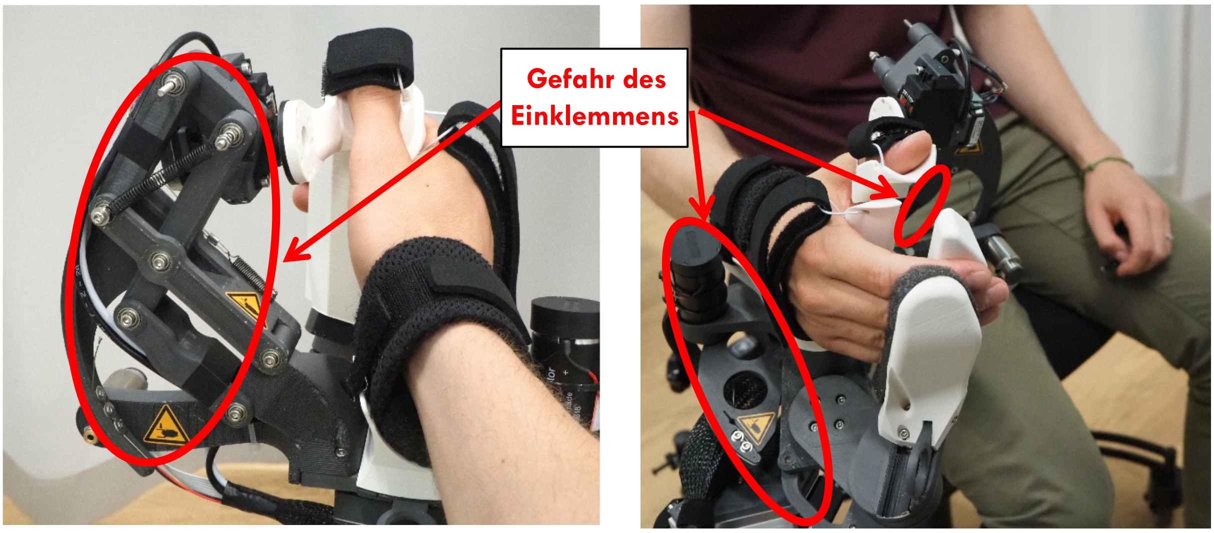

Figure 2 - Potenziell gefährliche Zonen am Handmodul

Motor Learning & Neurorehabilitation Laboratory
Kontakt: raphael.raetz@unibe.ch
Version: 14.03.2023 18:57
