# Supplementary material for: Enhancing stroke rehabilitation with whole-hand haptic rendering: development and clinical usability evaluation of a novel upper-limb rehabilitation device
Source: J Neuroeng Rehabil. 2024 Sep 27;21:172. doi: 10.1186/s12984-024-01439-1 (PMC11437669; doi:10.1186/s12984-024-01439-1)
Supplement: Supplementary file 2 [file 12984_2024_1439_MOESM2_ESM.pdf]

1 Questionnaires

Table 1: System Usability Scale (SUS) questions. The therapists completed two SUS questionnaires, where term *system* was replaced by *robot* and *graphical user interface (GUI)* respectively for finer granularity. Items that are inverted in the analysis are marked with (R).

**Nr.**	**Question**
Q1:	I think that I would like to use this *system* frequently.
Q2:	I found the *system* unnecessarily complex. (R)
Q3:	I thought the *system* was easy to use.
Q4:	I think that I would need the support of a technical person to be able to use this *system.* (R)
Q5:	I found the various functions in this *system* were well integrated.
Q6:	I thought there was too much inconsistency in this *system.* (R)
Q7:	I would imagine that most people would learn to use this *system* very quickly.
Q8:	I found the *system* very cumbersome to use. (R)
Q9:	I felt very confident using the *system.*
Q10:	I needed to learn a lot of things before I could get going with this *system.* (R)

Table 2: Perceived Usefulness (PU) questionnaire.

**Nr.**	**Question**
Q1:	Using the system in my job would enable me to accomplish tasks more quickly.
Q2:	Using the system would improve my job performance.
Q3:	Using the system in my job would increase my productivity.
Q4:	Using the system would enhance my effectiveness on the job.
Q5:	Using the system would make it easier to do my job.
Q6:	I would find the system useful in my job.

Table 3: Interest/Enjoyment subscale of the Intrinsic Motivation Inventory (IMI) questionnaire. Items that are inverted in the analysis are marked with (R)

**Nr.**	**Question**
Q1:	I enjoyed doing this activity very much.
Q2:	This activity was fun to do.
Q3:	I thought this was a boring activity. (R)
Q4:	This activity did not hold my attention at all. (R)
Q5:	I would describe this activity as very interesting.
Q6:	I thought this activity was quite enjoyable.
Q7:	While I was doing this activity, I was thinking about how much I enjoyed it.

2 Semi-structured Interviews

Table 4: Initial questions for the semi-structured interviews with the therapists.

Nr.	Question
Q1:	Describe your experience with the therapy system.
Q2:	What do you think about the games?
Q3:	What do you think about the fact that you could feel the objects?
Q4:	What do you think about the user interface?
Q5:	What do you think about the robot?
Q6:	What do you think about the movements that can be performed with the robot?
Q7:	How do you estimate the transferability to activities of daily living?
Q8:	What would you improve?
Q9:	Is there anything else you would like to say?

Table 5: Initial questions for the semi-structured interviews with the patients.

Nr.	Question
Q1:	Describe your experience with the therapy system.
Q2:	What do you think about the therapy exercises?
Q3:	What do you think about the fact that you could feel the objects?
Q4:	What do you think about the therapy robot?
Q5:	What would you improve?
Q6:	Is there anything else you would like to say?

3 Questionnaire Results

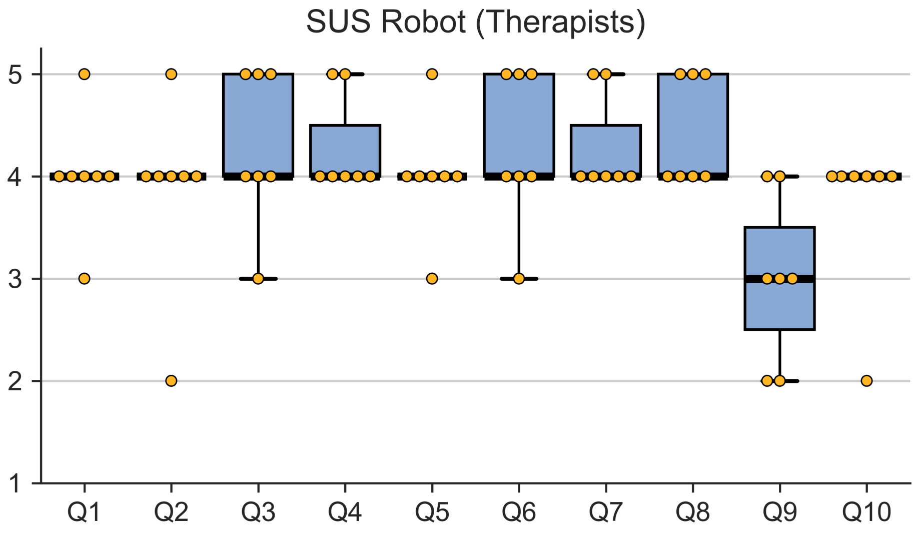

Figure 1: Therapists' responses to the individual questions of the SUS questionnaire on the robotic device. The five-point Likert items ranged from "Strongly disagree" to "Strongly agree".

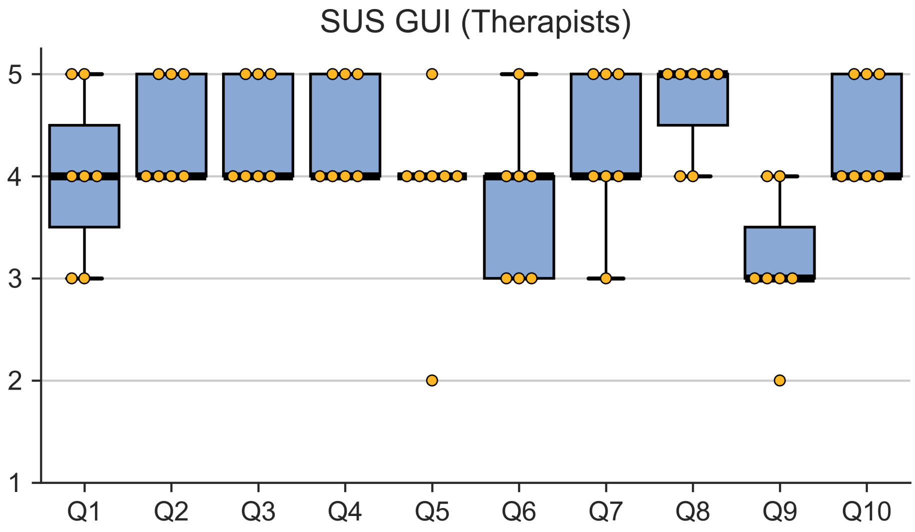

Figure 2: Therapists' responses to the individual questions of the SUS questionnaire on the graphical user interface. The five-point Likert items ranged from "Strongly disagree" to "Strongly agree".

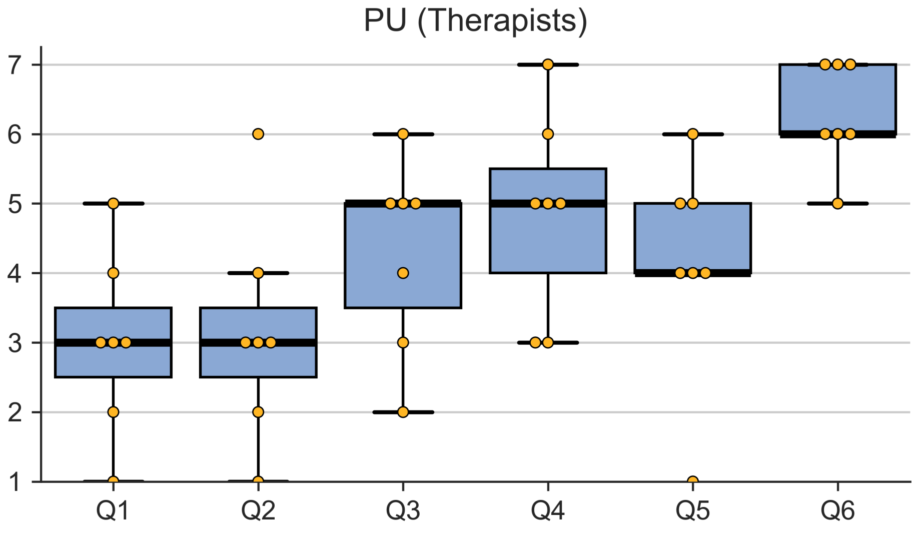

Figure 3: Therapists' responses to the individual questions of the PU questionnaire'. The seven-point Likert items ranged from 'Unlikely" to "Likely".

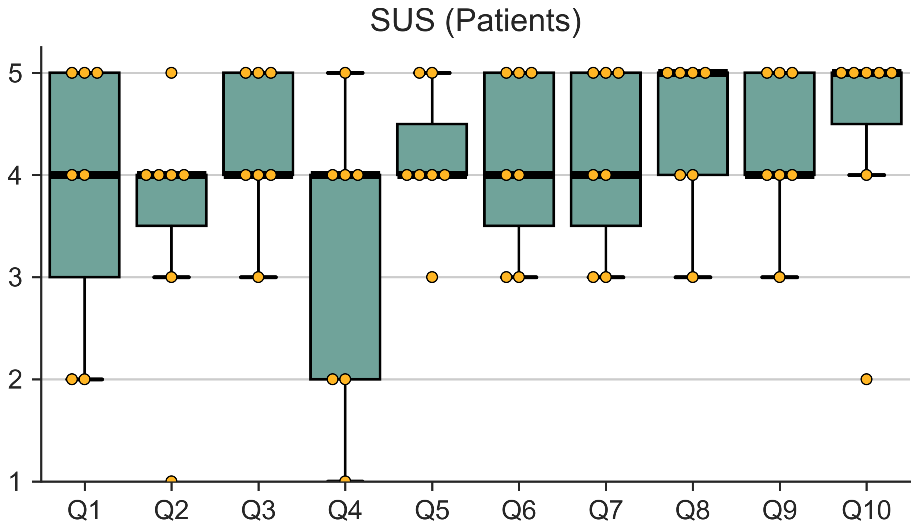

Figure 4: Patients' responses to the individual questions of the SUS questionnaire on the system. The five-point Likert items ranged from "Strongly disagree" to "Strongly agree".

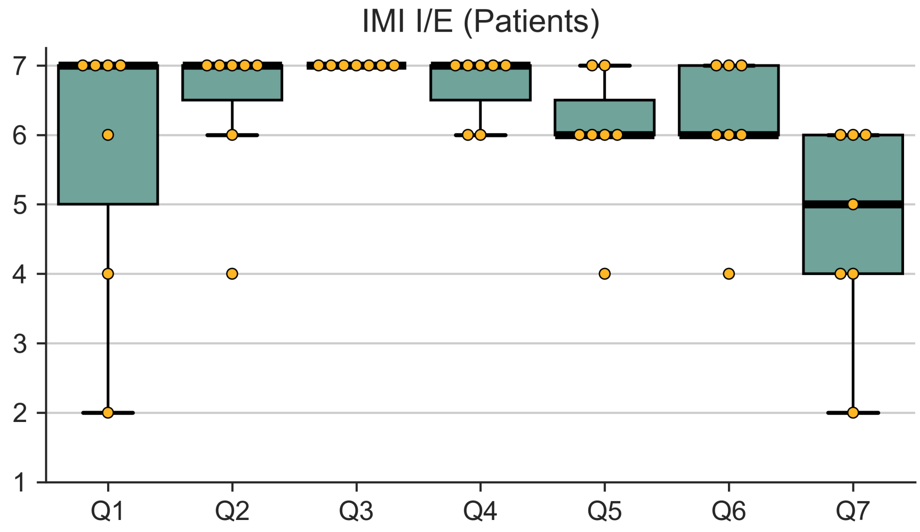

Figure 5: Patients' responses to the individual questions of the Interest/Enjoyment subscale of the IMI. The five-point Likert items ranged from "Not at all true" to "Very true".
